# Ethanol- and Cigarette Smoke-Related Alternations in Oral Redox Homeostasis

**DOI:** 10.3389/fphys.2021.793028

**Published:** 2022-01-28

**Authors:** Sara Zięba, Mateusz Maciejczyk, Anna Zalewska

**Affiliations:** ^1^Doctoral School, Medical University of Bialystok, Bialystok, Poland; ^2^Department of Hygiene, Epidemiology and Ergonomics, Medical University of Bialystok, Bialystok, Poland; ^3^Independent Laboratory of Experimental Dentistry, Medical University of Bialystok, Bialystok, Poland; ^4^Department of Restorative Dentistry, Medical University of Bialystok, Bialystok, Poland

**Keywords:** smoking, alcohol, oxidative stress, free radicals, salivary antioxidants

## Abstract

Alcohol abuse as well as smoking cigarettes has been proven to negatively affect the oral environment. The aim of this work was to provide a systematic review of the literature on the influence of ethanol and cigarette smoking on oral redox homeostasis. A search was performed for scientific articles indexed in the PubMed, Medline and Web of Science databases. We identified 32,300 articles, of which 54 were used for the final review, including the results from 2000 to 2021. Among the publications used to write this article, *n* = 14 were related to the influence of alcohol consumption (clinical studies *n* = 6, experimental studies *n* = 8) and *n* = 40 were related to the influence of smoking (clinical studies *n* = 33, experimental studies *n* = 7) on oral redox homeostasis. The reviewed literature indicates that alcohol abusers and smokers are more likely to suffer from salivary gland dysfunction, as well as develop precancerous lesions due to DNA damage. Compared to alcohol abstainers and non-smokers, alcohol drinkers and smokers are also characterized by a deterioration in periodontal health measured by various indicators of periodontal status. In summary, alcohol abuse and smoking are associated with disrupted oral redox homeostasis, which may lead not only to tooth loss, but also contribute to various adverse effects related to mental health, digestive processes and chronic inflammation throughout the human body.

## Introduction

Molecular oxygen is an essential element of life for aerobic organisms. However, its incomplete reduction leads to the formation of reactive oxygen species (ROS), such as hydrogen peroxide H_2_O_2_, superoxide radical anion (O_2_^⋅^), hydroperoxyl radical (HO_2_^⋅^), hydroxyl radical (^⋅^OH), nitric oxide (NO^⋅^) as well as singlet oxygen (^1^O_2_), peroxyl radical (LOO^⋅^), lipid hydroperoxide (LOOH), peroxynitrite (ONOO^–^), hypochlorous acid (HOCl) and ozone (O_3_) (Birben et al., 2012). At physiological concentrations, ROS play important functions in cells, including participating in intra- and intercellular signal transduction as well as exerting toxic effects on phagocytosed bacteria, parasites and tumor cells (Gołąb et al., 2002).

Aerobic organisms have developed mechanisms protecting them against the effects of an excessive supply of ROS and their reactions with cellular components. According to the definition: “antioxidants are compounds that, when present in low concentration compared to an oxidized substrate, can inhibit the processes of that substrate oxidation, scavenge or detoxify the already formed ROS, and repair ROS-induced damage of a target molecule” (Waddington et al., 2008). These include both low molecular weight antioxidants (uric acid, ascorbic acid, reduced glutathione (GSH), lactoferrin, amylase, thioredoxin, methionine) and enzymatic proteins (superoxide dismutase (SOD), catalase (CAT), peroxidase (Px), glutathione peroxidases (GPx), heme oxygenase, NADPH oxidoreductases).

The state in which the overproduction of ROS exceeds the body’s antioxidant capacity is called oxidative stress (OS). The effect of non-neutralized ROS can be temporary or even permanent changes in the structure of DNA, RNA, proteins, lipids and glycoconjugates. It has been demonstrated that oxidation of amino acid residues of a polypeptide chain leads to its defragmentation, formation of cross-links within one or between several protein chains, and modification of the amino acid itself (Stadtman and Levine, 2003; Ponczek and Wachowicz, 2005). A thoroughly researched free radical process is the phenomenon of lipid peroxidation. The biological consequence of lipid peroxidation is damage and depolarization of cytoplasmic and mitochondrial membranes: (Kalra et al., 1988, 1990; Kalra, 1989) and cell damage, as well as generation of further free radicals in the cell (Nigam and Schewe, 2000; Kang and Hamasaki, 2003; Knaś et al., 2012). ROS reactions with DNA result in damage to single nitrogen bases, breaks in the DNA strand and formation of adducts (Williams and Jeffrey, 2000; Cadet, 2003). These ROS effects at the cellular level result in cell death, accelerated ageing, neoplastic transformation and cell proinflammatory responses. These processes constitute the cellular basis for ROS- mediated diseases (Hopkins, 2017).

The oral cavity can be defined as the site of exchange between the body and the external environment, a site characterized by high complexity of ROS exposure. These exposures can result from ionizing and ultraviolet radiation as well as air and food pollution. Other sources of ROS in the oral cavity include medications, dental materials and treatment procedures, ongoing inflammation and bacteria (Waszkiewicz et al., 2012b; Zieniewska et al., 2020). Ethanol intake and smoking cigarettes are also significant sources of ROS. The latter two sources of exogenous ROS constitute a particular global problem in both health and economic terms. According to the WHO, people over 13 years of age consume approximately 6.2 L of pure ethanol/year, which amounts to 13.5 g of ethanol/day (WHO, 2014). Alcohol has been classified by the International Agency for Research on Cancer as a human carcinogen, and its consumption has been recognized as an increased risk factor for liver, breast, colorectal and upper aerodigestive tract cancers (Stornetta et al., 2018). Moreover, it is estimated that about 6–7% of global mortality is associated with alcohol use (WHO, 2014). Similarly alarming are the data on tobacco smoking. Globally, there are approximately 1.3 billion smokers and this number is predicted to rise to 1.7 billion by 2025 (Babizhayev and Yegorov, 2011). It should be emphasized that smoking causes the deaths of 5 million adults per year, including hundreds of thousands of so-called premature death cases (Babizhayev and Yegorov, 2011).

Although smoking can disrupt oral homeostasis, there is a lack of review summarizing current knowledge on the effects of alcohol and tobacco smoke on oral redox balance. Our manuscript is the first to systematically discuss the effects of ethanol and cigarettes on the antioxidant systems, oxidative and nitrosative stress, and mitochondrial function in the oral cavity.

It has been proven that OS may lead to morphological changes in the parenchyma of the salivary glands, which results in the decrease of the salivary secretion and biochemical changes in the saliva (Zalewska et al., 2014, 2015; Knaś et al., 2016; Kołodziej et al., 2017a,b). This state can lead to the development of other oral diseases such as xerostomia, burning mouth syndrome, periodontitis and precancerous lesions. Increased concentration of protein, lipids, and DNA oxidative damage markers, as well as changes of concentrations/activity of enzymatic and non-enzymatic antioxidants, were observed in the saliva and/or gingival fluid of patients with periodontitis, diabetes, insulin resistance and dementia (Choromańska et al., 2015; Wang et al., 2015, 2017; Zalewska et al., 2015; Knaś et al., 2016; Kołodziej et al., 2017a,b). Therapy with antioxidants has been suggested as a new therapeutic option for the above-named patients, however, sources of free radicals in the oral cavity are still not known exactly.

## Materials and Methods

### Search Strategy

The literature review was conducted from 2000 to January 2021 using the PubMed, Medline and Web of Science databases. The available literature was browsed based on the following keywords: [alcohol and ROS], [alcohol and salivary oxidative stress] [alcohol and periodontal tissue], [alcohol and cancer], [binge drinking], [smoking and salivary oxidative stress], [smoking and ROS], [smoking and oral cancer], [smoking and oral inflammation], [smoking and periodontal health]. The inclusion and exclusion criteria are presented in Table 1.

**TABLE 1:** Inclusion and exclusion criteria.

Inclusion criteria	Exclusion criteria
1. Articles written in English only. 2. Publications on the effects of salivary free radicals generated through smoking and alcohol consumption on oral health 3. Results obtained from experiments participated by humans as well as experimental works, including *in vitro* studies. 4. Meta-analyses.	1. Publications written in a language other than English. 2. Publications that did not evaluate the effects of salivary free radicals generated by smoking and alcohol consumption on oral health 3. Surveys. 4. Case descriptions.

### Data Extraction

The preliminary data extraction was performed based on the evaluation of titles and abstracts of manuscript independently by three researchers (SZ, AZ, MM). Then, texts of all the papers selected at the first stage of our work were reviewed and the studies meeting the inclusion and exclusion criteria were used for the final analysis. Determination of the reliability level of the researchers was performed using Cohen’s kappa coefficient (κ) which amounted to κ = 0.93. In order to ensure data quality, all publications were evaluated in terms of methodology and the following variables were distinguished: authors, year of publication, study design, size of the study population, inclusion and exclusion criteria, duration of the study and research results.

## Results

**FIGURE 1:**
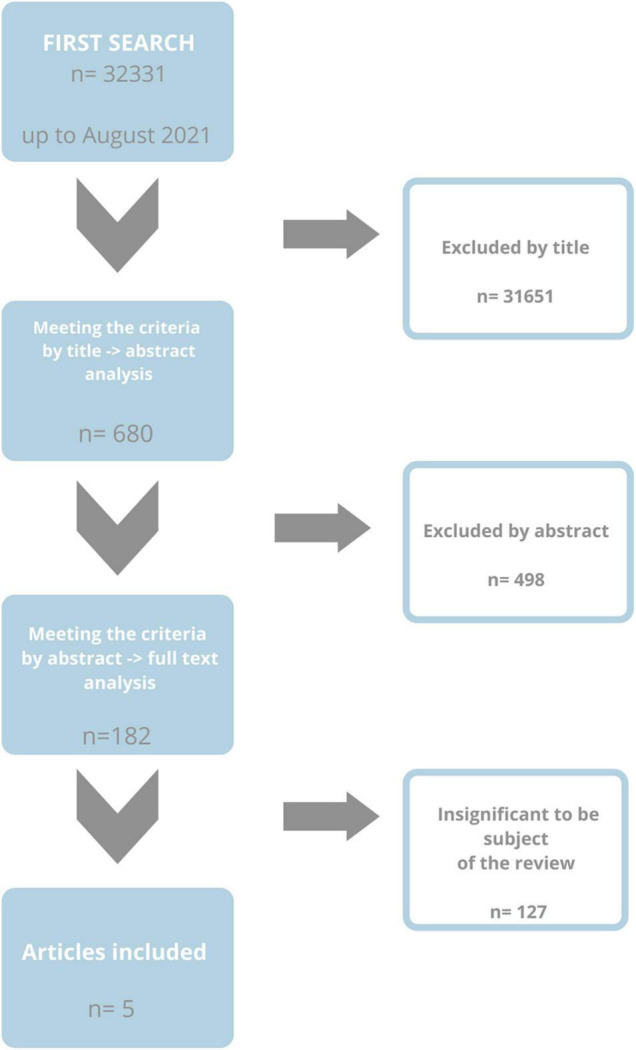
Flow chart of research methodology.

Of approximately 32,300 publications, 54 were classified as meeting the inclusion and exclusion criteria. The literature review revealed 32,331 works from the MEDLINE (PubMed) library, of which 31,651 were excluded due to the title. A total of 680 abstracts were read, 498 of which met the inclusion and exclusion criteria. Among the qualified articles, 128 turned out to be irrelevant for the subject of our review. Therefore, 54 papers were finally included (Figure 1).

### Alcohol and Reactive Oxygen Species

Ethanol has a form of non-ionized, small and chemically inert molecules. Due to these characteristics, it diffuses very easily into saliva and oral tissues after ingestion. Post-alcoholic damage to oral tissues results either from direct effects of ethanol or indirectly via its metabolites: acetaldehyde, reactive oxygen and nitrogen species (ROS, RNS) as well as fatty acid ethyl esters. The latter are non-oxidative metabolites of ethanol (Waszkiewicz et al., 2011).

The role of ethanol in terms of ROS/RNS, and thus oxidative stress, is ambiguous. Ethanol is one of the most effective “scavengers” of ^⋅^OH which is the most reactive oxidant able to oxidatively modify all substances present in the body. The human body does not have endogenous antioxidant systems capable of neutralizing this free radical (Halliwell and Gutteridge, 2006). However, for ethanol to neutralize ^⋅^OH we would have to consume it in quantities many times exceeding the lethal dose.

In the human body, ethanol is oxidized to acetaldehyde and then to acetate. This process is called ethanol intoxication. The former reaction is catalyzed mainly by alcohol dehydrogenases (ADH). It is worth mentioning that immediately after consumption, the concentration of ethanol in saliva is higher than in plasma. Half an hour after consumption, salivary concentration of ethanol is 10 times higher compared to its plasma level (Waszkiewicz et al., 2008b). The main metabolite of ethanol, acetaldehyde, reaches 10–40 times higher concentrations in saliva than in plasma, regardless of the ethanol dose ingested (Lachenmeier and Sohnius, 2008). Such high levels of acetaldehyde are caused by the intensification of ethanol metabolism in saliva as a result of ADH activity of the oral mucosa, as well as of bacterial ADH activity (Salaspuro, 2003). In the course of chronic alcoholism or in case of consuming large amounts of ethanol at a time, ADH are supported by microsomal processes of ethanol metabolism, which is undoubtedly affected by increased NADH reoxidation. Higher level of NADPH is a defense reaction of the body aimed at accelerating ethanol oxidation and eliminating it from the body (French, 1989). In microsomes, ethanol is oxidized by cytochrome P450, particularly by its isoenzyme 2E1 (CYP 2E1) and microsomal oxidoreductase independent of cytochrome P450, consuming iron ions (Dicker and Cederbaum, 1991). Furthermore, consumption of a high dose of ethanol at a time leads to a higher than normal one-electron oxygen reduction with the formation of O_2_^⋅^, which, in turn, damages mitochondria leading to hypoxia of the organ (Nordmann et al., 1992). Another enzyme that oxidizes ethanol is CAT which exhibits peroxidase properties in the presence of ethanol. Of the enzymes mentioned, only CAT – in the process of ethanol oxidation – does not produce ROS. On the contrary, it consumes H_2_O_2_. Alcohol dehydrogenase oxidizes ethanol with simultaneous formation of HO_2_^⋅^, while the reaction catalyzed by microsomal enzymes generates the most dangerous OH^⋅^ (Bondy, 1992)! By activating cytochromes CYP2E1 and salivary cytochromes CYP1A2 and CYP3A4 (Waszkiewicz et al., 2013) as well as undergoing enzymatic (xanthine oxidase and aldehyde oxidase) conversion to acetic acid, the resulting acetaldehyde increases the ROS pool in the form of O_2_^⋅^ and H_2_O_2_ (Niemelä, 2007).

Furthermore, ethanol has been demonstrated to promote the release of low molecular weight iron from bound intracellular reserves, which facilitates the Fenton reaction (Rouach et al., 1990). It can also induce the conversion of xanthine dehydrogenase to xanthine oxidase (Bondy, 1992).

### Binge Drinking Related Reactive Oxygen Species/Reactive Nitrogen Species and Saliva and Salivary Glands

According to the definition, 5 or more drinks for men and 4 or more for women, consumed within a 2-h period, are referred to as binge drinking. Such manner of ethanol intake results in blood concentrations of this alcohol of 0.08 g/dL or higher. Binge drinking is a major social problem as it leads to injuries, violence, chronic diseases, unwanted pregnancies, fetal alcohol spectrum disorders (FASDs) and other pregnancy complications (Waszkiewicz et al., 2008a). As demonstrated by, admittedly, few studies, this method of drinking results in salivary gland dysregulation and oral redox imbalance. Ferreira et al. (2021) investigated the effect of binge drinking during critical periods of pregnancy on the salivary glands of offspring in rats. Ethanol was administered during gestational days: GD6, GD7, GD8, i.e., during gastrulation and neurulation. The results of their study demonstrated that the parotid glands of the offspring of rats was more vulnerable to alcohol binge drinking. This greater damage was reflected in considerably intensified process of lipid peroxidation and decreased antioxidant capacity compared to the parotid glands of control group rats. In addition to boosted lipid peroxidation, the results of the ethanol-exposed group indicated a reduction in the peroxyl radical scavenging reaction in the parotid glands, which, according to the authors, appears to be one of the causes of the morphometric changes observed in the parenchyma of these salivary glands. Similar significant changes related to the phenomenon of lipid peroxidation were observed in the submandibular salivary glands, but they were approximately 1.5 times less severe than in the parotid glands. In contrast, the antioxidant capacity of the submandibular glands was not altered, which, according to the researchers, confirms the low intensity of OS in the cells of these salivary glands. The effect of episodic and intensive ethanol intoxication in a 3-day/week binge pattern on the redox balance of the parotid and submandibular glands of rats during their adolescence to young adult period showed a higher level of lipid peroxidation rate in the parotid glands of the ethanol group after 1 and 4 weeks of alcohol consumption (Fagundes et al., 2016). Submandibular glands of ethanol-treated group revealed considerably higher concentration of malondialdehyde (MDA) only after the first week of ethanol intake in a binge model. The lack of change during the further period of the experiment suggests adaptation of the glands to oxidative injury in a period of chronic binging. As suggested by the authors of both study groups, the observed differences in redox imbalance between the salivary glands of rats exposed to ethanol intake in a binge model were caused by the different morphology of the two glands as well as the fact that the parotid glands, unlike the aerobic submandibular glands, have an aerobic metabolism, which predisposes them to greater exposure and damage by ROS. In our study, we attempted to explain the effects of acute ethanol intoxication in occasional drinkers on antioxidant salivary proteins. We demonstrated that even a single ingestion of a relatively large but still tolerable dose of ethanol by young men significantly reduces the activity of peroxidase and minute secretion of lactoferrin in saliva, which undoubtedly affects the overall antioxidant capacity of the saliva (Waszkiewicz et al., 2012b). It is noteworthy that salivary peroxidase is one of the most important antioxidants synthesized by the salivary glands, although it constitutes only 0.01% of the total salivary protein content. Taking into account the antibacterial properties of peroxidase and lactoferrin, the observed reduction in their activity/minute secretion may predispose the oral mucosa to more frequent infections (Zalewska et al., 2013, 2014).

### Chronic Alcohol Intoxication Related-Reactive Oxygen Species/Reactive Nitrogen Species and Oral Health

Alcoholism has been recognized as a disease by the World Health Organization, and thus is included in the International Statistical Classification of Diseases and Related Health Problems (ICD-10) and the Classification of the American Psychiatric Association (DSM-V) as one of mental and behavioral disorders caused by the use of psychoactive substances. Alcohol dependence syndrome is a group of physiological, behavioral and cognitive phenomena, among which drinking alcohol dominates over other behaviors previously more important to the person. The main symptom of alcohol dependence syndrome is a loss of control over the amount of alcohol consumed and a constant need/desire to drink it (known as alcohol craving). Approximately 2% of the population exhibits chronic alcohol dependence (Waszkiewicz et al., 2012a,b,c). Devastating effects of alcohol dependence obviously include the negative consequences on oral health, thus also the redox balance of the salivary glands, mucosa and periodontal tissues.

### Ethanol- Derived Reactive Oxygen Species and Saliva and Salivary Glands

In the research of Campos et al. (2005) ethanol was applied to animals participating in the experiment in drinking water according to the model of moderate alcohol intoxication. Additionally, the authors decided to evaluate the effect of the exogenous antioxidant, α- tocopherol, in order to determine its influence on ethanol-associated salivary gland alteration. The observed changes in oxido-reductive impairment, as in the binge drinking models, depended on the type of salivary glands of rats.

The authors demonstrated increased activity of SOD and GPx in the homogenates of the parotid glands of ethanol-intoxicated rats, but no change in CAT activity. Simultaneous administration of ethanol and α-tocopherol caused these activities to drop to a level observed in the control group. Interestingly, none of the analyzed antioxidant enzymes revealed significant changes in the submandibular glands of ethanol-exposed rats as well as rodents exposed to ethanol combined with α-tocopherol compared to the control rats. The authors conclude that the observed changes in the activity of antioxidant enzymes in the parotid glands are due to the fact that this type of salivary gland has a greater abundance of antioxidant activity vs. the submandibular gland. The observed increase in enzyme activity may also be a defensive reaction of these glands to the increasing number of ethanol-derived ROS. Unfortunately, as further results of studies indicate, increased activity of antioxidant enzymes was not enough to counterbalance ROS, since significantly higher concentrations of thiobarbituric reactive substances (TBARS, products of lipid peroxidation) and carbonyl groups (products of protein oxidation) were observed vs. the control group. Administration of α-tocopherol considerably reduced the process of lipid peroxidation, while it could not inhibit the boosted process of protein oxidation in comparison with the group consuming ethanol. The effect of α-tocopherol on the process of lipid peroxidation in ethanol-treated group is most probably caused by the participation of this antioxidant in the synthesis of prostaglandins. It has been demonstrated that ethanol reduces prostaglandin synthesis in the sublingual and submandibular glands of rats (Wu-Wang et al., 1991), which proves the self-regulatory modulation of receptor binding induced by high ethanol concentration (Wu-Wang et al., 1992). α-tocopherol antagonizes inhibitory effect of ethanol on prostaglandin synthesis, thereby reducing the negative influence of ethanol on the parotid glands. Ethanol consumption in a model of moderate ethanol dependence did not induce oxidative stress in the submandibular glands of rats, as demonstrated by the lack of changes in the measurable results of lipid peroxidation and protein oxidation. The latter results in particular show that rat parotid glands are more damaged than the submandibular glands due to ROS activity generated by chronic alcohol consumption (Campos et al., 2005).

The study by Waszkiewicz et al. (2012b) indicated a significant increase in Px activity after chronic alcohol intoxication in subjects additionally addicted to nicotine in relation to the control group. Despite the well-known fact that ethanol induces changes in mucosa permeability, which facilitates the penetration of ROS/RNS from cigarette smoke, Waszkiewicz et al. (2012b) did not observe any additive effect of nicotine on Px activity. Moreover, they demonstrated a positive correlation between Px activity and the number of days of alcohol intoxication and no such correlation between Px activity and the number of cigarettes smoked or the duration of nicotine dependence.

According to the authors, these observations prove that the increase in Px activity is a consequence of chronic OS caused by ethanol action rather than smoking. On the other hand, higher Px activity may result from an influx of lymphocytes into damaged oral mucosa, as evidenced by increased periodontal indices (Waszkiewicz et al., 2012b). Moreover, based on the performed analyses, the authors suggest that salivary Px has a high diagnostic value connected with chronic alcohol abuse, as it is helpful in differentiating between alcoholics and the control group (Waszkiewicz et al., 2012b). However, acute ethanol intoxication in chronic drinkers significantly decreases the minute secretion of lactoferrin (Waszkiewicz et al., 2008b), glutathione S-transferase and total antioxidant capacity (TAC) of saliva (Peter, 2013). Reduced parameters of the aforementioned components of the antioxidant barrier prove a high increase in ROS production, which consistently utilizes antioxidants in ROS combating. Lactoferrin deficiency with accompanying inflammation may be a factor increasing iron bioavailability, which may lead to enhanced Fenton reactions and boosted hydroxyl radical production. OS process and its negative effects on the redox balance of saliva appear to be partially reversible once the source of ROS generation is removed. Peter (2013) demonstrated that only a one-month period of alcohol withdrawal significantly increases glutathione S-transferase and TAC, although not to the levels observed in the control groups.

### Oral Cancer and Precancer and Ethanol- Derived Reactive Oxygen Species/Reactive Nitrogen Species

The mechanism of ethanol-induced carcinogenesis is poorly defined; therefore, studies are being conducted to investigate it (Hecht, 2003). Warnakulasuriya et al. (2007) examined the generation and distribution of protein oxidation adducts with acetaldehyde and lipid peroxidation products: MDA and hydroxynonenal (HNE), as well as ethanol-stimulated expression of CYP2E1 in the oral mucosa of the alcohol-dependent patients diagnosed with leukoplakia (pre-cancerous condition) and squamous cell carcinoma (SCC). Positive staining toward the formation of protein adducts in oral tissues was observed in 61% for acetaldehyde, 57% for MDA and 80% for HNE in the dysplastic or cancerous biopsy specimens taken from the oral cavity. When the samples were differentiated into pre-neoplastic and neoplastic, MDA protein adducts were more marked in the first group. In contrast, patients with SCC were characterized by longer duration of addiction to alcohol. The authors also noted a positive correlation between the amount of alcohol consumed and CYP2E1 expression, which in turn was reflected in the intensity of tissue accumulation of protein adducts from HNE in both groups of patients. It was evidenced that the accumulation of ethanol-induced protein adducts in tissues reveals impairment of mechanisms aimed at removal of complex protein oxidation products. The accumulation of such adducts favors the formation of mutations and may lead to malignancy, as described previously (Guéraud, 2017). The authors postulate taking their results into account in studies on ethanol-induced carcinogenesis and in the comprehensive assessment and treatment of patients who excessively consume ethanol.

The results of Carrard et al. (2013) are contradictory to the above conclusions. These researchers demonstrated that increased cell proliferation with alcohol-related mucosa damage is not directly caused by the oxidative-reductive imbalance in tongue tissue. Interestingly, the authors did not observe OS symptoms in the form of increased concentration of oxidation products, but only changes in the activity of antioxidant enzymes and only in the group of rats exposed to alcohol for 60 days (decreased SOD activity, increased CAT activity). It is not surprising that increased CAT activity coexisted with decreased expression of nuclear factor E2-related factor 2 (NrF2), which is consistent with the previously demonstrated NrF2-independent mechanism of regulating the activity of this enzyme (Hopkins, 2017). NrF2 is a factor that regulates the expression of antioxidant genes and thus the antioxidant response. When bound to a cis-regulatory element, known as an antioxidant response, the element in the promoter region of antioxidant enzyme genes boosts the transcriptional expression (Hopkins, 2017). CAT is an enzyme without an antioxidant response element in its promoter center and it is not subjected to NrF2-mediated transcriptional activation. It is interesting to note that the authors observed a decrease in the concentration of lipid peroxidation products after 14 and 60 days of exposure to ethanol, which, according to them, is evidence of positive modification of cell membranes (Carrard et al., 2009, 2013). The keratinized layer of the tongue epithelium is a tissue consisting of extremely flattened and dehydrated cells. According to the authors, it is possible that high doses of ethanol enhance this protective layer, which has previously been described as “fixative,” and improve the epithelial permeability barrier (Du et al., 2000). At 120-day exposure, they observed normalization of all the evaluated OS parameters in the tongue mucosa to the control group levels. However, their findings indicate that the signal for cell proliferation, but not for an increase in alcohol-related epithelial proliferation, may result from elevated concentration of H_2_O_2_, which, according to them, is reflected by higher CAT activity at day 60 of exposure.

### Relationship Between Periodontal Disease and Alcohol-Derived Reactive Oxygen Species

The existence of a correlation between ethanol abuse and the development and severity of periodontal disease prompted a search for the effect of ethanol-induced ROS overproduction on periodontal tissues. Irie et al. (2008) examined the effect of chronic ethanol consumption on the periodontal tissue status and OS with and without ligature-induced inflammation in a rat model. Ethanol consumption increased 8-hydroxy-2′-deoxyguanosine (8-OHdG) concentrations, simultaneously reducing GSH/GSSG ratio in the “healthy”″ gingiva vs the control group. A similar pattern of results was obtained in the group of rats with induced periodontal disease and exposed to ethanol compared to the rat group with induced periodontal disease. It should be emphasized that although the reduction of GSH/GSSG ratio was at a comparable level in both groups exposed to ethanol, the concentration of 8-OHdG was higher in the group of ethanol-exposed rats with periodontal disease, which proves an addictive effect of this alcohol on gingival oxidative modification and destruction. This finding coincides with the evaluation of the effect of ethanol on periodontal tissue morphology. In the ethanol-exposed group with ligature-induced periodontitis vs. the group with periodontitis, only an influx of multinucleated lymphocytes into the gingival tissue was observed. Exposure of healthy rats to ethanol resulted only in apical migration of junctional epithelium, alveolar bone resorption and influx of multinucleated leukocytes into gingival tissue vs. healthy gingiva, but still more periodontal tissue destruction was observed in the ethanol plus periodontitis group vs. ethanol group. The results of Tomofuji et al. (2008) are also interesting, even though they do not directly involve the oral cavity. The authors demonstrated that the combination of ethanol and ligature-induced periodontitis caused higher concentrations of hexanoyl-lysine (HEL, lipid hydroperoxide-modified lysine residue formed at the early stage of lipid peroxidation) and 8-OHdG in the rat liver in comparison with ethanol exposure alone. The authors believe that periodontal diseases exacerbate the effect of ethanol on oxidative liver damage, particularly related to lipid peroxidation.

**FIGURE 2:**
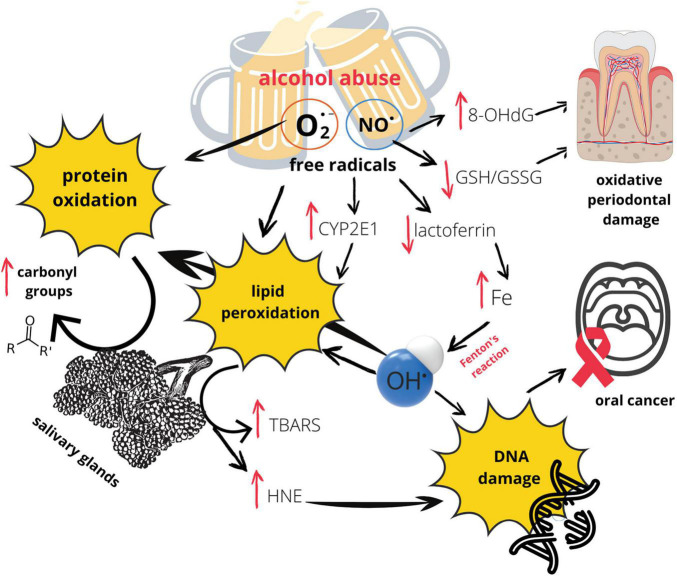
Effects of alcohol abuse on oral redox homeostasis. 8-OHdG, 8-Hydroxy-2′-deoxyguanosine; CYP2E1, Cytochrome P450 2E1; GSH/GSSG, glutathione/glutathione disulfide; HNE, 4 hydroxynonenal; O_2_^⋅–^, superoxide anion radical; NO^⋅^, nitric oxide; TBARS, thiobarbituric acid reactive substances.

Short-term ethanol (20%) consumption exerts addictive effect only on inducible nitric oxide synthase (iNOS) mRNA expression, but not on ligature-induced alveolar bone loss or on prostaglandin E2 concentration, both enhanced by periodontitis (Dantas et al., 2012). Moreover, the authors observed increased iNOS activity in the gingival tissue after short period of alcohol intake vs. healthy gingiva, which entails an increase in nitric oxide (NO) concentration in the gingiva. It was evidenced that NO promotes osteoclast maturation and enhances bone resorption induced by inflammatory cytokines (Brandi et al., 1995; Ralston et al., 2009). Additionally, ethanol-induced OS is one of the factors involved in the activation of the mammalian target of rapamycin (mTOR), which is essential to premature senescence in cementoblasts and human periodontal ligament cells (Bae et al., 2018). Therefore, the observed lack of changes in alveolar bone volume after exposure of healthy rats to ethanol is quite surprising, according to the authors. They explain their results by the short duration of the experiment and only 20% of ethanol concentration (Dantas et al., 2012). Graphic presentation of the influence of alcohol on oral redox homeostasis (Figure 2).

### Beneficial Effects of Red Wine Consumption

Research conducted over the past few years have proven the positive effect of polyphenols contained in red wine on oral health, obviously under conditions of moderate consumption of this type of alcohol. It should be mentioned that polyphenols are also contained in many other food products (herbs, spices, fruits and vegetables like apples, carrots, broccoli) as well as in green tea and their positive effect on redox homeostasis has been summarized in many other reviews (Tarascou et al., 2010; Gaur and Agnihotri, 2014; Wojtunik-Kulesza et al., 2020). Antioxidant capacity of polyphenols contained in grapes/wine is much stronger than that of vitamins C and E. One-electron reduction potential of epigallocatechin gallate is almost the same as of α-tocopherol, 550 and 480 mV, respectively (Frei and Higdon, 2003; Maeta et al., 2007). The antioxidant properties of polyphenols result, *inter alia*, from direct inactivation of ROS, activation of antioxidant enzymes (Nagata et al., 1999), ability to chelate metal ions (Chlebda et al., 2010), inhibition of oxidases (Hanasaki et al., 1994; Ursini et al., 1994) and increase in concentrations of uric acid and other low molecular weight antioxidants (Natella et al., 2002).

Varoni et al. (2013) evaluated salivary antiradical capacity and total polyphenol content after consumption of wine (125 mL) and then after 4 weeks of total abstinence 300 mg of non-alcoholic red wine extract (minimum 95% polyphenols). The results of their study indicated that wine consumption (18% ethanol) did not significantly reduce salivary antiradical capacity, which could result from the antioxidant properties of polyphenols counteracting the pro-oxidant properties of ethanol. The authors argue that the lack of increase in salivary antioxidant capacity after wine consumption (presence of antioxidants!) is caused by the possible pro-oxidant properties of polyphenols which attach to the oral mucosa and may undergo oxidation (Varoni et al., 2013). Wine extract increased the antioxidant properties of saliva very efficiently, which was most likely related to the presence of the polyphenols themselves, although their concentration in saliva after wine intake was negligible. Varoni et al. (2013) suggest that polyphenols from the extract, but not from the wine, increase uric acid and glutathione production via unknown mechanisms. The suggestion is too far-fetched, as the authors did not determine salivary concentrations of the aforementioned antioxidants, which could have confirmed their assumption. The researchers observed higher amounts of polyphenols remaining in the oral cavity after the consumption of wine than of the extract, although it should be highlighted that polyphenols from wine were deprived of their antioxidant properties (they were used in scavenging of ethanol-induced ROS). Melatonin contained, for instance, in red wine is characterized by a rich spectrum of activities, including antioxidant and anti-inflammatory effects. It is believed that melatonin can act as a potent antioxidant enhancing mechanisms counteracting oral diseases (Balaji et al., 2015). Unfortunately, according to a study by Varoni et al. (2018), consumption of red wine with high melatonin content did not significantly increase its salivary concentration. However, the authors emphasize numerous limitations of their experiment (the need for versification of red wine melatonin isomers, need for additional measurements of daily salivary melatonin concentrations, which would help to better understand how wine can modify the circadian rhythm of endogenous melatonin) that may potentially influence the obtained results.

Red wine is rich in polyphenols, but also has a very low pH comparable to orange and apple juice, which makes it a beverage of high erosion potential. Carvalho et al. (2021), evaluating the erosion kinetics of red wine and apple as well as orange juice in the presence and absence of acquired pellicle, demonstrated that in both experiments red wine showed the highest surface reflection intensity (lowest degree of erosion of the polished enamel surface) and the highest surface hardness, followed by orange and apple juice. Moreover, the authors observed a nearly linear correlation between the polyphenol content of the tested beverages and intensity of tooth enamel erosion: the higher the polyphenol concentration, the smaller the erosive demineralization. The anti-erosive effect of red wine is most likely caused by polyphenol-induced acquired pellicle modification. According to the authors, polyphenol molecules bind with salivary proteins to form protein-polyphenol complexes that build into the acquired pellicle. Once attached to the pellicle, they form a scaffold to which other salivary proteins can easily attach. Evidence has shown that the protein content of the salivary pellicle modified by red wine consumption is approximately 10 times higher than in the control plate, making it thicker and more resistant to removal (Joiner et al., 2003, 2004; Sierpinska et al., 2013). However, the described pellicle modification occurs only in case of frequent and slow red wine drinking (the drink stays in the mouth for a long time). Such a pellicle provides better protection against low pH of red wine, as evidenced by slight changes in enamel hardness before and after its consumption. The authors underline that white wine, containing about 100 times less polyphenols than red wine, has truly high erosive potential.

NO is not only a free radical involved in pathophysiological processes in the oral cavity, as described above. As a signaling molecule, NO plays very important roles in physiological processes. It has been demonstrated that moderate consumption of nitrate-rich fruit and vegetables is a natural and low-cost way to stay healthy. According to the authors of the experiment, the increase in salivary NO (Takahama et al., 2010) obtained after mixing white wine with saliva is therefore a positive sign. It can increase eradication of microorganisms (Xu et al., 2001) and oral mucosal thickness (Björne et al., 2004), as can be observed in the stomach.

Physiological NO concentrations are also essential in the regulation of oral blood flow (Toda et al., 2012) and proper salivary gland secretion (Modin et al., 1994). It should be mentioned, that saliva is for the oral cavity the same as blood is for the whole body. The correct flow of saliva determines the hydration of the mucosa and provides nutrients and components of specific and non-specific immunity, and thus may have a preventive effect against mucositis and oral infections.

### Smoking and Reactive Oxygen Species

Traditional cigarettes are a source of massive amounts of ROS/RNS. The source of the generated ROS/RNS is the cigarette smoke (CS) produced during smoking. CS consists of a main stream (inhaled by the smoker at the time of drawing on a cigarette) and a secondary stream produced while burning the tobacco. The latter poses danger both to the smoker and the environment.

The main stream can be divided into a gas phase and a solid phase, or cigarette tar (a residue that remains on a filter that traps 99.9% of particles with a diameter of over 0.1 micrometer after passing cigarette smoke through) (Valavanidis et al., 2009). The amount of cigarette tar produced depends on the quality of a given cigarette. However, it is estimated that smoking an average cigarette weighing 1 g leads to the formation of 20 mg of tar.

The quality and quantity of ROS/RNS present in the gas and solid phases differ. Tobacco tar provides huge amounts of ROS (about 10^17^ per gram of tar) as well as heavy metals and carcinogenic organic compounds. The main representatives of cigarette ROS are stable free radicals quinone, semiquinone, hydroquinone (Q/QH•/QH_2_) (Pryor et al., 1990; Pyror, 1992). These compounds are in mutual balance and have a long half-life. Extraction of the tar phase in aqueous solution leads to the formation of O_2_^⋅–^, H_2_O_2_ and, as mentioned above, the most dangerous ^⋅^OH in the following: the QH• radicals reduces O_2_ into O_2_^⋅–^, which can dismutate to form H_2_O_2_ and then with Fe2 + can generate through the Fenton reaction highly oxidizing hydroxyl radicals (Pryor et al., 1983a; Valavanidis et al., 2009). Moreover, ROS of the solid as well as gas phase are responsible for the release of iron from the endogenous enzyme ferritin, and may thus enhance the production of ^⋅^OH in the aforementioned Fenton reaction. It is interesting to note that concentrations of tar-like substances in cigarettes is similar to that in exhaust particles of diesel engines (Ross et al., 1982). The above-mentioned heavy metals included in cigarette smoke, especially cadmium play an important role in the production of oxygen free radicals (Genchi et al., 2020). Its toxic effect is mainly due to the blockage of the mitochondrial electron transfer chain by impeding the flow of electrons through mitochondrial complex III (main place of production of ROS) (Checa and Aran, 2020). Cadmium changes the activity of mitochondrial proteins by inhibiting the enzymes of the respiratory chain, which in turn leads to mitochondrial swelling and impairment of cellular respiration (Cannino et al., 2009). In addition, it can lead to the release of cytochrome c from the mitochondrion, which is synonymous with the activation of the caspase pathway and the initiation of the process of cellular apoptosis (Checa and Aran, 2020; Genchi et al., 2020). It is estimated that the gas phase of one drag of a cigarette delivers about 10^15^ RO with a low reactivity period into the body (Pryor et al., 1983b). Despite the short reactivity of individual radicals, their high concentration in cigarette smoke persists for more than 10 min. ROS/RNS in the gas phase are formed continuously, and their concentration in the oral cavity increases with the duration of the addiction to smoking. An important role in the generation of free radicals in the gas phase is played by nitric oxide (NO) (Pryor and Stone, 1993) which is responsible for the initiation of a cascade of oxidation reactions. Significant amounts of NO can be found in smoke and therefore, despite its low reactivity, it undergoes constant oxidation to the much more reactive nitrogen dioxide (NO_2_). In the next stage, NO_2_ reacts with other components of cigarette smoke such as isoprene to form free radicals with an unpaired electron on the carbon atom and peroxyl and alkoxy radicals.

As in the main stream, the side-stream smoke consists of a gas and a solid phase. The secondary stream of CS is estimated to contain even 170 times more ammonia, five times more carbon monoxide and ten times more hydrogen cyanide or nitrogen oxides than the CS main stream. The ROS generated by the secondary smoke are highly reactive despite their short duration period. These implications mean that the passive smoker unknowingly “smokes” several cigarettes a day and inhales massive amounts of ROS generated by the active smoker. It has been evidenced that passive smoking is responsible for serious health consequences for non-smokers, making the secondary smoke even more harmful (Valavanidis et al., 2009).

### Cigarette Addiction and Oral Health

#### Saliva, Salivary Glands and Cigarette-Related Reactive Oxygen Species/Reactive Nitrogen Species

The oral cavity is the first place where cigarette smoke comes into contact with the human body, and this fact that has an impact on its health. A large panel of papers evaluating the antioxidant capacity of smokers’ saliva is available. The results of most studies clearly indicate that smoking cigarettes is accompanied by decreased activity of endogenous salivary enzymatic antioxidants such as SOD, CAT and Px as well as reduced concentration of non-enzymatic endo- and exo-antioxidants: GSH, UA (Nagler) and vitamin C (Klein et al., 2003; Reznick et al., 2003; Nagler, 2007; Abdolsamadi et al., 2011; Falsafi et al., 2016; Arbabi-Kalati et al., 2017; Singh et al., 2018). According to the available evidence, cigarette smoke disturbs the metabolism of trace elements which are cofactors of antioxidant enzymes (copper and zinc as cofactors of SOD, iron as cofactor of CAT), thus reducing their activity (Nobari et al., 2021). It is noteworthy that a significant reduction in salivary Px activity was observed in a group of non-smokers after smoking one cigarette (Reznick et al., 2003). Smoking one cigarette led to a greater decrease of Px activity in the non-smoking group compared to the smoking group. The authors suggest a protective role of higher concentrations of SCN^–^ ions in the saliva of compulsive smokers. It has been proven that the addition of exogenous SCN^–^ to the saliva of non-smokers “immunized” to some extent, but only temporarily, salivary Px against damaging effects of cigarette smoke, which is attributed to the extended biological half-life of the molecule (9.5 h) (Azen, 1978; Reznick et al., 2003). However, the exact mechanism of such “protection” has not been demonstrated. Reduced activity of the aforementioned antioxidant enzymes is probably the reason for the significant TAC reduction/TOS increase observed in numerous studies (Charalabopoulos et al., 2004; Abdolsamadi et al., 2011; Bakhtiari et al., 2015; Neshat et al., 2020). Importantly, it was stated in several papers that TAC was significantly lower in the saliva of passive smokers that in active smokers (Azadbakht et al., 2016; Neshat et al., 2020) as well as that TAC in passive smokers was significantly lower compared to non-smokers (Mottalebnejad et al., 2014; Nobari et al., 2021). Nobari et al. (2021) evaluated the effect of intense physical exercise, the side effect of which is extremely intense OS, on the activity of SOD, CAT and Px in groups of physically active and passive subjects exposed and not exposed to cigarettes. The researchers demonstrated that intense training significantly increases SOD, CAT and Px activity, which the authors believe is a positive response to the increasing amount of ROS. However, in the smoking groups, the activity of the said enzymes was lower than in the non-smoking groups. The authors conclude that the smoking habit attenuates the good effect of daily physical activity on acute exercise-induced OS.

It has been demonstrated that even a 3-week exogenous vitamin C supplementation is unable to elevate TAC levels in smokers in whom these levels remain similar to the status before the supplementation period (Bakhtiari et al., 2012). However, consumption of green tea improves the salivary TAC in smokers. Azimi et al. (2017) measured the salivary TAC of smokers at the baseline, then after 7 and 21 days of drinking two cups of green tea per day. At day zero, the non-smoking group had statistically significantly higher TAC. Although drinking green tea did not considerably increase salivary TAC in the smoking group to the levels observed in the control group after 7 and 21 days, there was an upward trend in both groups. Regular drinking of black tea has been shown to have an even better effect on the oral antioxidant defense system. In a study by Pal et al. (2012) smokers with a tea drinking habit were characterized by lower levels of ROS and lower DNA damage in exfoliated buccal cells. Moreover, the habit of drinking tea effectively decreased the expression of ROS-generating proteins – I_K_B (I kappa B kinase), NF-_K_B (nuclear factor kappa-light-chain-enhancer of activated B cells), DNA-associated proteins p53 (tumor protein p53) and MLH1 (gene mutS HOMOLOG 1) in tobacco users. Activation of NF-_K_B is responsible, among others, for stimulation of osteoclasts and increased activity of metalloproteinases, resulting in overproduction of lipid peroxides, oxidized proteins and inflammatory factors, and ultimately leading to oxidative damage of tissues.

When antioxidant systems fail and the redox balance shifts toward oxidation, an increase in the concentration of salivary lipid peroxidation products generated by non-enzymatic pathways is observed (Guentsch et al., 2008; Demirtaş et al., 2014; Metgud and Bajaj, 2014; Kurku et al., 2015). In the study by Demirtaş et al. (2014), salivary MDA content in smokers was found to be significantly higher compared to the control group and the group of passive smokers, which was partially compliant with the findings of other research groups (Guentsch et al., 2008; Kurku et al., 2015). It has been proven that salivary MDA plays a pathological role in multistep oral carcinogenesis and progression. Metgud and Bajaj (2014) observed significant elevation of salivary MDA, rising progressively form healthy controls to pre-cancerous conditions and individuals with SCC, in line with a state of OS connected with SCC. In the saliva of compulsive smokers and subjects who were not smokers on a daily basis but who were exposed to smoking one cigarette for the purpose of the experiment, Reznick et al. (2003) observed significantly increased carbonylation of salivary proteins, which is a form of their oxidative damage. A similar increase in the concentration of carbonyl groups of proteins in the saliva of tobacco addicts, the so-called heavy smokers, immediately after smoking a cigarette was obtained by Nagler (2007). Proteins of great importance for maintaining not only the redox balance, but also the immunological and microbiological balance of saliva, such as amylase, acidic proline-rich proteins and lysozyme, were carbonylated. A significant reduction in bioavailable reduced disulfide groups of proteins was observed in the saliva of heavy smokers, but only directly after smoking a cigarette, compared to non-smokers (Kurku et al., 2015). This fact was associated by the authors with a significant decrease in GSH activity whose main role in the saliva is to maintain the disulfide groups of proteins in a reduced state, which determines their biological activity.

The significantly reduced gamma-glutamyltransferase activity in the saliva of smokers vs non-smokers, reported in the study by Greabu et al. (2007), may lead to the accumulation of harmful substances in the tissues of the oral cavity and salivary glands and to the development of numerous pathologies. The mentioned enzyme catalyzes the reaction of GSH with electrophilic substances. The products of the reaction are glutathione S-conjugates, and their formation is one of the steps of removing xenobiotics, including lipid peroxidation products, from the body.

#### Oral Cancer and Pre-cancerous and Cigarette-Related Reactive Oxygen Species/Reactive Nitrogen Species

It should be emphasized that in a situation of reduced Px and CAT activity, intoxication with H_2_O_2_ in the oral cavity is significantly reduced. In the presence of metal ions, either from the parotid glands or directly from cigarette smoke, H_2_O_2_ initiates the previously described Fenton and Haber Weiss reactions, leading to the formation of ^⋅^OH (Nagler et al., 1997). It is worth remembering that mitochondrial DNA (mtDNA) is particularly sensitive to the effect of ^⋅^OH. The amount of ^⋅^OH -related damage to mtDNA has been found to exceed the level of damage to nuclear DNA of the same cell by 10 times (Karolkiewicz, 2011). This is attributable to the presence of the respiratory chain in the mitochondria and the absence of histones, i.e., proteins that protect DNA from damage. At the same time, it has been observed that the ability to repair mtDNA and proteins associated with oxidative phosphorylation and damaged due to replication errors is limited (Pędzik et al., 2008; Ścibior-Bentkowska, 2009). Increased levels of oxidative DNA damage have been found to lead to reduced synthesis as well as activity of enzymes that remove these lesions, which is associated with increased cancer incidence (Halliwell, 2006; Greabu et al., 2009; Zaremba, 2010). Oxidative modifications of mtDNA are among the causes of oral squamous cell carcinoma (SCC) (Banerjee et al., 2017), and the incidence of oral SCC in smokers is four to seven times higher than in non-smokers. Naturally, one cigarette does not cause SCC, particularly since activity of, for instance, Px in the oral cavity returns to 90–100% of its initial level 30 min after smoking a cigarette, which is due to its continuous synthesis in the parotid glands (Reznick et al., 2003). However, according to the studies, compulsive smokers are firstly exposed to gradual accumulation of the damaging effects of damage caused by free radicals, which is most likely caused by the dysfunction of systems eliminating them (Isik et al., 2007; Caliri et al., 2021). In addition, the continuous daily deficit of H_2_O_2_-detoxifying enzymes in the saliva of compulsive smokers (who smoke 20 or more cigarettes per day on average) does not sufficiently protect the oral epithelium (Nagler et al., 1999). As a result, lesions are formed which are initially dysplastic, but over time may develop into a full-blown carcinoma *in situ*. These hypotheses are supported by the findings of Wu et al. (2005) who discovered that nicotine-induced DNA damage in oral cancer cell line was initiated by ROS.

Although the results quoted below were determined in the saliva, due to the fact that they are related to neoplastic conditions, we discuss them in the present subsection.

In terms of SCC development or pre-cancerous conditions in the oral cavity, the excess of NO delivered with cigarette smoke also appears to be of great significance (Preethi, 2016). It should be stressed, however, that the determination of NO in the saliva is controversial because it is a very unstable compound and is transformed into numerous nitrogen derivatives, including its most dangerous derivative – peroxynitrite. This might explain the results of Nagler who demonstrated a significant decrease in NO in the saliva of heavy smokers. Similar findings were obtained by Kanehira et al. (2006), but these authors observed a significant increase in NO concentration in the saliva of smokers immediately after smoking a cigarette, both in comparison with the control group and in the saliva of smokers before smoking a cigarette. It is believed that overproduction of NO may contribute to carcinogenesis or other pathological processes in the oral cavity in two ways: via its direct effect on biological targets (short-term effect), or indirectly – through the generation of peroxynitrite. It has been proven that peroxynitrite is involved in the pathogenesis of oral cancer through the formation of carcinogenic nitrosamines or inhibition of the DNA repair mechanism (Cortés-Ramírez et al., 2009). In addition to the above-mentioned functions, GSH is also used as a cofactor by glutathione peroxidase in the neutralization of peroxynitrite (Hopkins, 2017). The aforementioned reduction in GSH concentration in the saliva of smokers vs non-smokers may enhance OH. formation, thus increasing the ROS and peroxynitrite load (Zappacosta et al., 2002). It should be noted there are doubts concerning the source of NO in the saliva of smokers in the literature. Authors wonder whether elevated salivary NO concentrations contribute to the development of pre-cancerous lesions or pre-cancerous lesions contribute to increased levels of Preethi (2016).

The results of a study on the effect of smoking on the function of the 18 kDa translocator protein (TSPO) are also important in the terms of oral cancer development (Nagler et al., 2010). TSPO is an intracellular protein located mainly on the outer mitochondrial membrane. It is present in almost all tissues (mainly, however, in those with high energy requirements) and its presence has been found in the cell fraction of human saliva (Nagler et al., 1999). TSPO protein plays a role, *inter alia*, in the regulation of apoptosis. Interestingly, depending on the type of ligand, it can induce apoptosis as well as exhibit anti-apoptotic effects (Veenman et al., 2007, 2008). The exposure of saliva to cigarette smoke free radicals results in a three tines lower affinity of salivary TSPO to its specific ligand PK 11195. It should be mentioned that PK 11195 is responsible for cell cycle arrest in the G1/S phase and induces cell apoptosis (Mendonça-Torres and Roberts, 2013). These observations indirectly suggest that PK 11195 ligand expression decreased by TSPO may lead to the development of malignant lesions, including oral cancers, due to inhibition of apoptosis and, consequently, uncontrolled division of damaged cells.

Depletion of salivary antioxidant defense was undoubtedly not always observed when exposed to ROS increased by cigarette smoke. Kanehira et al. (2006) observed higher SOD activity in non-stimulated saliva of older smokers (65 years old or more) compared to their non-smoking peers, which agreed with Nagler’s (2007) results in the saliva of older heavy smokers immediately after smoking a cigarette. According to Kanehira et al., the elevated SOD activity results from adaptive mechanisms that develop in older adults in response to dysfunction of mitochondrial respiratory chains. Mitochondrial dysfunction is one of the manifestations of aging. Mitochondria are the main cellular site of ROS generation. Studies have revealed an age-related dysfunction of respiratory-chain complex I and increased production of O_2_^–^, which naturally increases the activity of dismutases (Martínez-Cisuelo et al., 2016). In both aforementioned studies, other antioxidant enzymes, CAT and Px, are not efficient in neutralizing excess H_2_O_2_, which consequently promotes OH^–^ generation. Moreover, Nagler (2007) showed that salivary antioxidant capacity (ImAnOx) of older heavy smokers was significantly higher compared to non-smokers, which was an unsuccessful attempt to neutralize ROS/RNS – unsuccessful, because, as mentioned above, the authors noted increased carbonylation of salivary proteins, which is a sign of OS.

There are reports demonstrating no significant changes in enzyme activities, TAS/TOS levels, concentrations of oxidation products in the saliva of young moderate smokers compared to the control group (Charalabopoulos et al., 2004; Mottalebnejad et al., 2014; Kurku et al., 2015; Neshat et al., 2020). The authors believe that this lack of changes is caused by great compensatory capacity of young organisms to the increasing ROS/RNS levels and the short duration of addiction of the examined subjects.

#### Cigarette- Born Reactive Oxygen Species/Reactive Nitrogen Species and Periodontal Diseases

Secondary to bacterial plaque, smoking cigarettes is one of the major determinants of the onset and progression of periodontal disease. The effects of periodontal diseases include not only tooth loss, social alienation, psychological injuries related to disturbed facial aesthetics and pronunciation, as well as digestive processes (Coelho et al., 2020). It is primarily a chronic inflammation that spreads throughout the body through the bloodstream. It has been proven that periodontal disease is associated with arthritis and other autoimmune diseases as well as cardiovascular disease, diabetes and obesity (Keller et al., 2015; Kaur et al., 2017; Makkar et al., 2018).

Components of tobacco smoke contribute to the dysfunction and oxidative damage of human gingival fibroblast (HGF), consequently leading to progressive failure of gingival connective tissue. In a study by Colombo et al. (2012) exposure of HGF to cigarette smoke had a lethal effect within a short period of time. Interestingly, the authors suggest that the decreased viability of HGF was not caused by cell apoptosis (microscopic examination did not reveal the presence of any apoptotic bodies typical of this process, and no condensation of cell nuclei or fragmentation of DNA/perinuclear bodies was observed).

The authors suggest that one of the possible reasons for the reduced viability of HGF is the occurrence of abnormalities in their cellular morphology. Smoking increases the concentration of intracellular ROS and carbonylation of proteins, including cytoskeletal proteins such as actin (the formation of actin filaments plays a major role in shaping and maintaining cell stability) and cofilin-1 (by mediating actin filament crossing and depolymerization/remodeling, it is responsible for cytoskeleton dynamics). Oxidative modification of cytoskeletal proteins results in impairment of the motility, adhesion and division of fibroblast cells (Hotulainen et al., 2005). In addition, a CS exposure dose-dependent decrease of reduced cellular protein thiols and intracellular GSH in HGF/PDLF (periodontal ligament fibroblast) has been reported, which partly coincides with the results of Chang et al. (2002). The noted result is not surprising in light of the above facts, as the main function of GSH is to maintain a reduced state of the thiol groups of proteins. Previously, studies showed that human gingival fibroblasts rapidly absorb and accumulate high levels of nicotine *in vitro*. The absorbed nicotine remains inside the fibroblasts, where it can affect cell metabolism or function (Hanes et al., 1991; Chang et al., 2002). To determine the mechanism of cytotoxicity of nicotine on PDLF, Chang et al. incubated cells with nicotine in medium and treated them with SOD, CAT, 2-oxothiazolidine-4-carboxylic acid (OTZ) and sulfoximine buthionine (BSO). Although SOD and CAT did not inhibit nicotine-induced cytotoxicity, the application of OTZ (a cysteine precursor that promotes GSH synthesis) led to cytotoxicity inhibition. In contrast, the addition of BSO (which is an inhibitor of cellular GSH synthesis) enhanced the cellular cytotoxicity of nicotine. Therefore, it appears that the cytotoxic effect of nicotine in PDLF culture was caused by GSH deficiency. Similarly, Tinti and Soory (2012) evaluated mechanisms for redox capacity of nicotine and GSH in HGF and human periodontal fibroblasts (HPF). This study involved a novel use of dihydrotestosterone (DHT) as an indicator of oxidative stress and wound healing (HGF and HPF cells exhibit high expression of the androgen receptor). Through the activation of androgen receptors, DHT has the ability to induce anti-apoptotic protein and to alleviate H_2_O_2_-induced oxidative stress. DHT shows the ability to activate CAT and suppress protein kinases (including NF-κB) (Giretti and Simoncini, 2008; Lee et al., 2008). Nicotine was shown to significantly decrease DHT activity. A similar report of nicotine dose-dependent OS, including in human periodontal ligament fibroblasts (PDLF) cells, was published by Nguyen et al. (2019). The authors demonstrated that oxidized guanine species (Ox-Gs) in PDLF lysate increased with an increase in nicotine concentration. Oxidative stress induced by *Porphyromonas gingivalis* lysate was also evaluated in this study. An interesting finding is the occurrence of significantly higher Ox-GS concentration in PDLF cultures exposed simultaneously to *P. gingivalis* and nicotine after 24 h, compared to cell cultured in serum-free medium served as a negative control. As the authors observed no differences after 2 h of exposure, they concluded that the effect of nicotine on periodontal tissues depends not only on its dose but also on the time of exposure. Another noteworthy conclusion of the study is the additive effect of nicotine and *P. gingivalis* on OS formation in periodontal tissues. Lipopolysaccharides present in *P. gingivalis* stimulate polymorphonuclear neutrophils (PMN) to phagocytosis, thus contributing to the “respiratory burst” process during which free radicals are generated. Moreover, nicotine metabolites, exerting effects similar to the activity of toxins produced by periopathogens, lead to the accumulation of free radicals and boost OS in periodontal tissues.

The expression of oxidative stress-related genes is thought to be induced particularly after exposure to compounds which react with thiols (Heikkila et al., 1982). Nicotine has been proven to be responsible for up-regulation of heme oxygenase-1 (HO-1) expression in human gingival fibroblasts (Chang et al., 2005). HO-1 acts as an oxidative enzyme by catalyzing the oxidative degradation of heme to biliverdin, which is then reduced to bilirubin – a compound with antioxidant properties (Vile and Tyrrell, 1993). In the study by Chang et al. (2005) HGF exposure to 10 mM nicotine induced HO-1 protein expression *in vivo* in a time-dependent manner. It should be mentioned that the regulation of nicotine-induced HO-1 expression depends on intracellular GSH concentration (Chang et al., 2005). The addition of SOD and CAT did not decrease HO-1 induction. After adding NAC – a GSH precursor – the authors observed a significant decrease in HO-1 expression compared to HGF challenged only with nicotine. Furthermore, smoking-induced free radical imbalance leads to the induction of cyclooxygenase-2 (COX-2) mRNA expression in HGF (Chang et al., 2003c). COX-2 is an enzyme responsible for the synthesis of prostaglandins as well as initiation of an inflammatory cascade (Chang et al., 2003b,a). Nevertheless, it has been demonstrated that the use of OTZ resulted in an approximately 60% reduction in nicotine-induced COX-2 protein levels in HGF (Ho and Chang, 2006). Similarly, application of PD98059 (an inhibitor of protein kinase regulated by an extracellular signal) led to a significant decrease in nicotine-induced levels of COX-2 in HGF, whereas the addition of BSO resulted in approximately three times higher levels of COX-2 protein. These data suggest that the oxidative effect of nicotine mediates COX-2 induction in HGF, and thiol pools may act as an intracellular buffer against nicotine-induced COX-2 expression.

Corrêa et al. (2019) evaluated the influence of resveratrol on oxidative stress-induced experimental periodontitis in rats exposed to cigarette smoke. Resveratrol is a molecule with anti-inflammatory and antioxidant properties. Furthermore, it inhibits the synthesis of the CYP450 enzyme responsible for ROS production and is an antagonist of the receptor of aryl hydrocarbon which also leads to increased free radical generation (Leonard et al., 2003; Deng et al., 2014). This study revealed a reduction in bone mass loss in rats exposed to cigarette smoke inhalation + resveratrol (SMK + RESV) compared to the control group (cigarette smoke inhalation + placebo/SMK + PLAC). Resveratrol supplementation in the SMK + RESV group resulted in increased SOD activity and reduced NADPH oxidase activity in periodontal tissues compared to the SMK + PLAC group. Moreover, in the study by Andreou et al. (2004) resveratrol demonstrated a pronounced antagonism to aryl hydrocarbon receptor (which has an oxidative effect) in rat bone marrow cells, and Ikeda et al. (2018) observed reduced bone loss and promotion of bone healing by systemic treatment with melinjo seed extract rich in resveratrol derivatives. It can be assumed that the above correlations are also due to the antioxidant effect of resveratrol, and thus the reduction of osteoclast activity.

Tonguç et al. (2011) demonstrated the additive effect of smoking and periodontitis on OS severity and reduction of antioxidant systems. The group of smokers with periodontitis had the highest level of MDA and the lowest activity of SOD, CAT and GSH-Px in gingival tissues compared to the control group of non-smokers with healthy periodontium.

Changes in the antioxidant capacity of smokers with periodontitis can also be monitored in saliva, although the available results on this subject are contradictory. Buduneli et al. (2006) observed no significant differences in a TAC level in NWS of smokers with periodontitis. Agnihotri et al. (2009) reported a significant decrease in SOD activity in the NWS and gingival fluid of smokers with periodontal disease compared to the control group (non-smokers with periodontal disease), which is also confirmed by the results of Bizoń and Milnerowicz (2014). The reduction in SOD activity was more visible in GCF than in saliva. The authors explain this observation by higher concentration of ROS in the GCF unit, which is lower compared to the volume of saliva in the mouth (the total non-stimulated saliva flow rate is approximately 0.3–0.4 ml/min, whereas for GCF it is several microliters per hour). In addition to “resource depletion,” cigarette-induced ROS may inactivate SOD through oxidative modifications of its protein chains as well as via modification of its activity caused by impaired metabolism of trace elements that are its cofactors. Yadav et al. (2020) compared metallothionein (MT) concentrations in NWS and GCF of smokers and non-smokers with chronic periodontitis as well as those with healthy periodontium. MT is an antioxidant protein rich in metal-binding cysteine. It is effective particularly against hydroxyl radicals (Thornalley and Vašák, 1985). MT content in the saliva of smokers with periodontitis was significantly higher compared to the other three groups. Furthermore, no difference in MT concentration was observed between non-smokers with periodontitis and smokers without any periodontal disease, which again proves an additive effect of cigarette radicals on periodontal disease progression. A similar correlation was observed in the study by Katsuragi et al. (1997) who demonstrated significantly higher MT concentration in the layer of gingival spinous epithelial cells in smokers with periodontitis compared to non-smokers with periodontitis. Interestingly, increased MT synthesis may down-regulate Cu^2^ + /Zn^2+^ SOD expression in the course of a compensatory mechanism (Nzengue et al., 2011). This is due to free radicals-induced MT-transcriptional responses through metal regulatory transcription factor 1 (MTF-1) which in the presence of Zn ions (displaced from SOD by cadmium contained in cigarette smoke), binds to a metal-responsive element in the promoter region of the MT gene to initiate its transcription (Andrews, 2000). Alteration in the activity of salivary cigarette-formed antioxidants results in increased concentrations of biomolecule oxidative modification products in saliva. In a study by Celec et al. (2005), TBARS and MDA levels were considerably higher in smokers with periodontitis compared to healthy controls. It is noteworthy that the study presented no significant correlations between TBARS in saliva and plasma of patients with periodontopathy. Therefore, it can be concluded that systemic oxidative stress does not directly affect salivary TBARS levels, and markers of lipid peroxidation in smokers with periodontitis are valuable indicators of exposure to ROS. Ips (8-epi-PGF2α, among others) are isomers of prostaglandins, formed in the process of enzymatic peroxidation of polyunsaturated fatty acids. In the study by Wolfram et al. (2006), the concentration of 8-epi-PGF2α in the NWS of smokers with periodontitis was significantly higher compared to non-smokers with periodontitis. Varghese et al. (2020) showed higher levels of salivary 8-OhdG in the group of smokers with chronic periodontitis compared to non-smoking controls with periodontitis. Salivary 8-OhdG levels correlated with clinical indicators of periodontal disease (plaque index, gingival index, pocket probing depth and clinical attachment levels). These correlations confirm that oxidative modification of bio-molecules, in this case DNA, may be one of the mechanisms of periodontal tissue destruction in the course of periodontitis. Unfortunately, non-surgical treatment consisting in scaling and root planning (SRP) performed in both groups did not lead to a reduction of 8-OhdG levels in the smoker group to the levels observed in healthy controls. A similar lack of effect of SRP on ascorbic acid levels in the NWS of smokers with periodontitis was demonstrated by Mathias et al. (2014). Contrary results were obtained Hendek et al. (2015) who determined the levels of 8-OhdG, 4-hydroxynonenal (HNE) and GPx activity in gingival fluid and saliva collected at the beginning as well as in the first and third month after SRP. The authors found that after non-surgical periodontal treatment, 8-OhdG concentration in GCF and saliva decreased significantly in both periodontitis groups (however, it was still higher in the smoking group compared to non-smokers), while the content of 4-HNE and GPx enzyme activity in GCF and saliva did not change significantly after the treatment in any of the study groups. In contrast, Guentsch et al. (2008) found that non-surgical periodontal treatment in smokers reduced MDA and GPx concentrations to levels comparable to healthy controls, both smokers and non-smokers. These results clearly indicate that periodontal therapy may be helpful in diminishing OS in smokers with periodontitis.

In light of changing trends connected with smoking, electronic cigarettes have been introduced to the market. These are devices that heat special inhalation solutions that may or may not contain nicotine (Korfei, 2018). Although most of long-term effects of e-cigarettes are still unknown due to the short presence of these devices on the consumer market, recent findings suggest that e-cigs may induce oxidative stress and increase the expression of advanced glycation end products (AGEs) and their cellular receptors (RAGE), also in gingival and periodontal tissues (Javed et al., 2017; Al-Aali et al., 2018; AlQahtani et al., 2018; ArRejaie et al., 2019). The increased number of AGE-RAGE bonds has been linked to the formation of reactive oxygen species that induce the oxidative burst of periodontal tissues and cause functional changes in polymorphonuclear chemotaxis and phagocytosis and increased systemic and local inflammatory burden through elevated cytokine expression in serum and gingival fluid. In addition, Ganapathy et al. (2017) demonstrated that exposure to aerosol extracts of e-cigs may lead to significant DNA damage in oral epithelial cells expressed with high concentrations of 8-oxo-dG. Analyzing the mechanisms that modulate e-cigarette-induced DNA damage, the authors identified an increase in ROS concentration, and a decrease in TAC and the expression of proteins essential for repairing oxidative DNA damage (8-oxoguanine DNA glycosylase, OGG1). These results clearly indicate that exposure of the oral cavity to electronic cigarette aerosol may potentially increase the risk of developing oral cancer.

**FIGURE 3:**
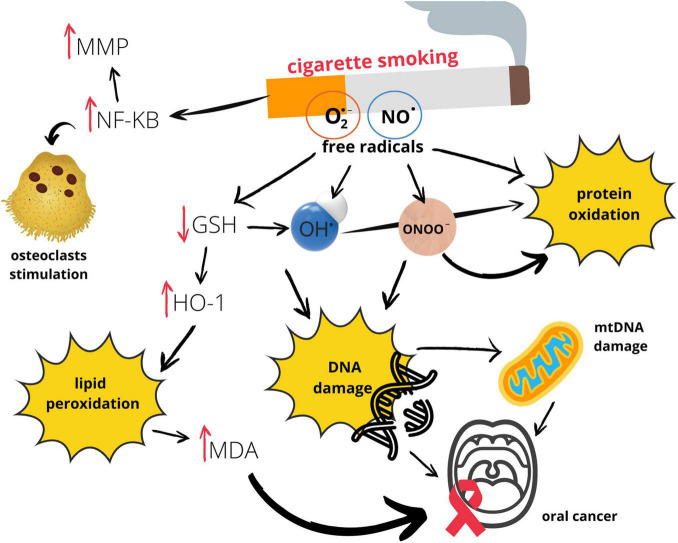
Effects of smoking on oral redox homeostasis. GSH, glutathione; HO–1, heme oxygenase 1; MDA, malondialdehyde; MMP, metalloproteinase; mtDNA, mitochondrial DNA; NF-_K_B, nuclear factor kappa-light-chain-enhancer of activated B cells; NO^⋅^, nitric oxide; O_2_^⋅–^, superoxide anion radical; OH^⋅^, hydroxyl radical; ONOO^–^, peroxynitrite.

Graphic presentation of the influence of smoking on oral redox homeostasis (Figure 3).

## Conclusion

Alcohol abuse and smoking have been substantial public health problems for years. Numerous studies have demonstrated that those habits cause damage to almost every organ of the body, including impairing the function of the oral cavity. As a result of excessive alcohol consumption and smoking, the oxidative balance in the oral cavity might be disturbed. Smoking and alcohol abuse might be reflected in reduced oral antioxidant capacity and in an increasing oxidative damage to cellular elements. Overproduction of free radicals might leads to DNA damage which increased risk of developing oral cancer. Alcohol and smoking habits might worsen the course of the already existing periodontal disease, which could not only be the cause of tooth loss, but could be responsible for the deterioration of mental health, leads to poorer digestion of food and, most importantly, could be associated with systemic inflammation. Unfortunately, what should be emphasized, the effect of exogenous antioxidants on strengthening the endogenous antioxidant barrier of the oral cavity of smokers and alcohol abusers are inconclusive.

What should be keep in mind, alcohol abuse and smoking, not least through redox imbalances may lead to the deterioration of the oral cavity homeostasis. The other factors, such as dehydration of the mucosa, its irritation, worse oral hygiene in addicted people, may contribute to the development of the discussed pathologies in the oral cavity. Similarly, the innate/hereditary antioxidant predisposition of the organism of alcohol abusers/smokers may influence the amount of generated oxidative stress in response to the above stimulants. Unfortunately, we are not able to carry out a meta-analysis, due to the large variety of publications (experimental and clinical publications). It is necessary to inform the public about the possible adverse effects of excessive alcohol consumption and smoking to raise awareness of the possible oral health consequences.

## Author Contributions

SZ: conceptualization, data curation, formal analysis, funding acquisition, investigation, methodology, writing—original draft. MM: conceptualization, investigation, writing—review and editing. AZ: conceptualization, data curation, formal analysis, funding acquisition, investigation, methodology, writing—original draft, writing—review and editing. All authors have read and agreed to the published version of the manuscript.

## Conflict of Interest

The authors declare that the research was conducted in the absence of any commercial or financial relationships that could be construed as a potential conflict of interest.

## Publisher’s Note

All claims expressed in this article are solely those of the authors and do not necessarily represent those of their affiliated organizations, or those of the publisher, the editors and the reviewers. Any product that may be evaluated in this article, or claim that may be made by its manufacturer, is not guaranteed or endorsed by the publisher.
