# Supplementary material for: Ethanol- and Cigarette Smoke-Related Alternations in Oral Redox Homeostasis
Source: Front Physiol. 2022 Jan 28;12:793028. doi: 10.3389/fphys.2021.793028 (PMC8832011; doi:10.3389/fphys.2021.793028)
Supplement: Supplementary file 1 [file Table_1.docx]

**Table 1. Publication significant to be subject of the review**. **AA** - ascorbic acid; **CAT** – catalase; **COX-2** – cyclooxygenase-2; **EC** - electronic cigarette; **GPx** - glutathione peroxidase; **HGF** - human gingival fibroblasts; **HPF** – periosteal fibroblast;**iNOS** - inducible nitric oxide synthase; **PDLF** - periodontal ligament fibroblasts; **GSH/GSSH ratio** – reduced/oxidized GSH; **LDH** - lactate dehydrogenase; **MDA** – malondialdehyde; **MLH1** - gene mutS HOMOLOG 1; **MT –** metallothionein; **NAC** - N-acetyl-1-cysteine; **NADPH** - nicotinamide adenine phosphatase oxidase; **NF-_Κ_B** - nuclear factor kappa-light-chain-enhancer of activated B cells; **OTZ** - oxothiazolidine carboxylate; **BSO** - DL-buthionine-(S,R)-sulfoximine; **PG** – parotid glands; **PDLF** – Periodontal Ligament Fibroblast Cells; **PGE-2** - prostaglandin E2; **PGF2-α** - prostaglandin F2 alpha; **Px** – peroxidase; **ROS** - reactive oxygen species; **SCC** - squamous cell carcinoma; **SLG** – sublingual glands; **SMG** - submandibular glands; **SOD** - superoxide dismutase; **TAC** - total antioxidant capacity; **TBARS** - thiobarbituric acid reactive substances; **UA** - uric acid; **H_2_O_2_**- hydrogen peroxide.

| Aim of the study | Population surveyed (n) | Studied material | End points | Authors |
| --- | --- | --- | --- | --- |
| *Alcohol* | | | | |
| **Clinical studies** | | | | |
| Determining the effect of chronic alcohol intoxication on the concentration and production of **lactoferrin** in saliva. | The patients (n=20) were divided into two groups:  A. non-smoking men after chronic alcohol intoxication (n=10).  C. non-smoking men who drink for social purposes (n=10). | **Unstimulated saliva** collected 24 to 48 hours after chronic alcohol intoxication. | Despite the no significant difference in the concentration of lactoferrin in saliva between the two groups, there was a significantly reduced production of lactoferrin in group A compared to group C.  Moreover, a significant correlation was found between the amount of alcohol consumed daily and the decrease in the production of lactoferrin. | (Zalewska et al., 2014) |
| Evaluating the effects of chronic alcohol intoxication and smoking on **Px** activity. | The patients (n=37) were divided into two groups:  1.male alcohol-dependent smokers (n=17).  2. male non-smokers and social drinkers controls (n=20). | **Unstimulated saliva** | There was a significantly higher Px activity in alcohol-dependent smokers compared to the control group. Px activity significantly correlated with the number of days of alcohol consumption, but not smoking. | (Waszkiewicz et al., 2012c) |
| Determining the influence of ethanol consumption on activity of salivary **Px.** | Healthy men (n=8) who did not meet any alcohol abuse criteria. | **Unstimulated saliva** collected at the start of the study and then 12, 36 and 108 hours after 6 hours of ethanol consumption. | 36 hours after drinking alcohol, the activity of peroxidase significantly decreased compared to the values obtained 12 hours before drinking alcohol.  Between 36 and 108 hours after consuming alcohol, peroxidase activity tended to increase, although at 108 hours after the drinking session, the peroxidase activity was still significantly lower than before drinking.  There was no significant change in lactoferrin levels after the drinking session. | (Waszkiewicz et al., 2008b) |
| Evaluating the effect of alcohol withdrawal on activity of salivary **GST** as well as **amylase** and **TAC** levels. | The patients (n=120) were divided into two groups:  1. alcohol-dependent men who were admitted to rehab for one month (n=60).  2. non-drinking control group (n=60). | **Unstimulated saliva** | Alcoholism is responsible for impaired antioxidant capacity and a reduction in amylase secretion, which can be partially reversed through alcohol withdrawal. People addicted to alcohol showed significantly lower GST, amylase and TAC activity in saliva compared to the control group. Alcohol withdrawal resulted in a significant increase in GST and amylase activity and TAC to values close to control. | (Peter, 2013) |
| Evaluating the protective effects of **polyphenols** contained in red wine/red wine extract on oral health. | Healthy adults (n=12) | **Unstimulated saliva** | The results indicate that drinking red wine does not cause increased spider activity in saliva. However, administration of red wine extract led to increased scavenging of free radicals. | (Varoni et al., 2013) |
| Investigating the distribution of **protein adducts** with **acetaldehyde** and lipid peroxidation end products **(MDA, HNE)** from patients with oral pre-cancer and cancer and reported alcohol abuse. | Two groups were distinguished in the study (n=36):  1. patients with oral leukoplakia (n=7).  2. patients with SCC (n=29). | Oral **biopsy specimens** | The results indicate that adducts derived from acetaldehyde and lipid peroxidation are formed in the oral tissues of alcohol abusing patients with oral leukoplakia and cancer. | (Warnakulasuriya et al., 2007) |
| **Experimental Studies** | | | | |
| Evaluating the biochemical and morphological effects of ethanol binge drinking during pregnancy on PG, SMG and saliva of offspring rats. | Pregnant *Wistar* rats (n=8) were divided into two groups:  1. Ethanol group (n=4).  2. Control group (n=4). | **PG, SMG** and**stimulated saliva** | PG of off springs rats was more vulnerable to binge drinking alcohol. This resulted in a significant increase in the lipid peroxidation process and a reduction in antioxidant capacity compared to the parotid glands of rats in the control group.  In the SMG of off spring rats, an increase in lipid peroxidation was also observed (1.5 times less intense than in the case of the parotid glands), however, the antioxidant capacity of the submandibular glands did not change. | (Ferreira et al., 2021) |
| Evaluating the effects of episodic and intense intoxication of ethanol in a 3-day/week binge drinking on the PG and SMG of rats, during adolescence to young adulthood phase. | *Wistar* rats (n=26) received ethanol (20%w/v) for 3 consecutive days/week (from the 35th until the 62nd day of life) simulating a pattern of binge consumption. After that animals were divided into four groups (n=5–8 animals per group):  1. 1 week of 3 days of exposure to ethanol.  2. 1 week of 3 days of exposure to distilled water.  3. weeks of 3 days of exposure to ethanol.  4. weeks of 3 days of exposure distilled water. | **PG** and **SMG** | Submandibular glands of ethanol treated group showed a significantly higher concentration of MDA only after the first week of ethanol intake in a binge model. Lack of changes in the further period of the experiment suggests the adaptation of the PG to oxidative injury in a period of chronic binging. According to the suggestions of the authors of both research groups, the observed differences in redox imbalance between the salivary glands of rats exposed to the intake of ethanol in a binge model were caused by the different morphology of both glands. | (Fagundes et al., 2016) |
| Determining the effect of chronic ethanol consumption on the oxidative status of rat’s PG and SMG by identifying the activities **SOD**, **CAT** and **GPx**. To estimate ethanol-associated glandular damage α -tocopherol was supplied to the animals and **TBARS** as well as **protein carbonyl** content were evaluated. | *Wistar* rats (n=24) were divided into two groups:  1. Ethanol group (n=12).  2. Control group (n=12). | **PG** and **SMG** | Significant increase in the activity of SOD and GPx was observed in homogenates of the PG of rats intoxicated with ethanol, with no changes in the CAT activity Simultaneous administration of ethanol and α- tocopherol caused these activities to drop to a level observed in the control group. Chronic ethanol treatment caused higher content of TBARS and protein carbonyl in PG. Supplementation of α-tocopherol suppressed the oxidative ethanol-induced damage in lipid without affecting induced protein oxidation  None of the analyzed antioxidant enzymes/TBARS/protein carbonyl content showed significant changes in the SMG of rats exposed to ethanol, as well as to ethanol in combination with α-tocopherol compared to the group of control rats. | (Campos et al., 2005) |
| Evaluating the influence of different concentrations of ethanol (0-5%) on content of three prostaglandins **PGE2, PGF2- α, 6-keto-PGF1- α**. | Adult *Sprague-Dawley* rats (n=10). | **SLB** and **SMG** | Alcohol impairs the function of the salivary glands by inhibiting the production of prostaglandins. In the sublingual gland, ethanol decreased the levels of PGE2 and PGF2-α , but had no effect on 6-keto-PGF1 α, while in the submandibular gland there was a significant reduction in all three prostaglandins. | Wu-Wang et al., 1991) |
| Determining whether oxidative stress parameters (**lipid peroxidation, protein carbonyls, SOD and CAT activity**) are implicated in the induction of cell proliferation in rat tongue epithelium after different times of chronic alcohol consumption. | *Wistar* rats (n=48) were divided into six groups:  1. control (n=6)  2. alcohol/60 days (n=10)  3. alcohol/120 days (n=10)  4. Tween (n=6)  5. Tween + vitamin E (n=6)  6. Tween + vitamin E + alcohol/120 days (n=10) | rats **tongue** epithelium | There was no differences in lipid peroxidation when comparing group 3 or 6. In the group 3 TBARS levels decreased when compared to the group 1 and 6. There wereno effects on protein carbonyls observed in the group 2, 3 and 6. Group 3 was characterized by lower activity of SOD and higher activity of CAT when compared to other groups .This results suggests that increase in the proliferation of rat tongue epithelial cells due to alcoholic damage is not directly related to an imbalance in oxidative conditions. The study showed that H_2_O_2_ is likely involved in cell signaling during proliferation in the oral mucosa. 60-day alcohol consumption changes the parameters of oxidative stress, but does not change cell proliferation. | (Carrard et al., 2013) |
| Evaluating the effect of acute alcohol consumption and concomitant vitamin E treatment on the parameters of oxidative stress **(TBARS, protein oxidative damage, CAT activity, SOD)** | *Wistar* rats (n=38) divided into five groups  1.alcohol (n=6).  2.alcohol / vitamin E (n=10).  3. control (n=6).  4.Tween (n=6).  5.vitamin E (n=10). | rats **tongue** | The results of the study indicate that even short-term alcohol consumption reduces the level of lipid peroxidation. In the alcohol group, there was a significant reduction in TBARS compared to the control group. Animals treated with alcohol-vitamins characterized a significant decreased in TBARS compared with the Tween and vitamin E groups. Moreover, the activity of CAT was significantly higher and SOD was significantly lower in the group of animals treated with both alcohol and vitamin E. | (Carrard et al., 2009) |
| Evaluating the influence of ethanol on periodontitis induction in rats by determining the content of **GSH/GSSH, 8-OHdG and TNF-α.** | *Wistar* rats (n=26) were divided into 4 groups (6-7 rats per group):  1. rats with ethanol diet.  2. rats control diet.  In one of each dietary group, periodontitis was ligature-induced, while the other group was left unligated. | Samples of the left **mandibular molar regions.** | Chronic consumption of ethanol led to a decrease in the GSH/GSSH ratio and increased the levels of 8-OHdG and TNF-α in the gingiva. The results of the study indicate that chronic alcohol consumption is responsible for the worsening of periodontitis and oxidative damage to the gums. | (Irie et al., 2008) |
| Determining the short term effect of ethanol administration on periodontal disease in rats by evaluating **mRNA** expression and **iNOS**activity. | Wistar rats were divided in four groups (n=24):  1. rats receiving water (n=6).  2. rats receiving ethanol (n=6). 3. rats submitted to experimental periodontitis and receiving water (n=6).  4. rats submitted to  experimental periodontitis and receiving ethanol (n=6). | **Gingival tissue** and half of the rats**lower jaw** | Induced periodontitis significantly increased mRNA expression and iNOS activity in the gingival tissue. Brief administration of ethanol had an additive effect on mRNA expression and iNO activity, enhanced by inflammation. Ligation of periodontitis, unlike the alcohol-only group, increased prostaglandin E(2) content and decreased alveolar bone volume compared to control rats. | (Dantas et al., 2012) |
| *Smoking* | | | | |
| **Clinical studies** | | | | |
| Evaluating the effect of smoking on activity of salivary  **UA, SOD, GPx, Px**. | The patients (n=80) were divided into two groups:  1. smoking men (n=40)  2. non-smoking control (n=40) | **Unstimulated saliva** | The mean levels SOD, GPx, and Px in unstimulated saliva were significantly lower in smokers than non-smokers. There was no statistically significant difference in the salivary UA level between smoking and non-smoking men. | (Abdolsamadi et al., 2011) |
| Determining antioxidant activity ( reflected in **SOD** and **GPx**activity) in saliva of tobacco users. | Healthy individuals divided in three groups (n=75):  1. (n=25): no periodontal  disease and never smoked.  2. (n=25): no periodontal disease, never used smokeless tobacco, but had 3 years history of smoking at least 10 cigarettes daily.  3. (n=25): no periodontal disease and had 1 year history of chewing a 10-g tobacco packet daily. | **Unstimulated saliva** | There were significant differences in the activities of salivary SOD and GPx among the three groups. Activity of GPx was higher in smokeless tobacco users. However, SOD activity was higher in non-tobacco users than users. | (Arbabi-Kalati et al., 2017) |
| Investigating for **CAT** activity and **α-amylase** levels in saliva of smoking individuals. | The patients (n=400) were divided into two groups:  1.active smokers (n=200).  2.nonsmokers (n=200). | **Unstimulated saliva** | There were no significant differences in the activities of salivary CAT and α -amylase levels among the smokers and nonsmokers group. | Singh et al., 2018) |
| Evaluating the **Px** activity in saliva prior to and after smoking one cigarette. | *In vivo study*  The patients (n=33) were divided into two groups:  1. smokers (n=17).  2. nonsmokers (n=16).  *In vitro study*  The patients (n=18) were divided into two groups:  1. smokers (n=7).  2. nonsmokers (n=11). | **Unstimulated saliva** | After smoking one cigarette a significant decrease in Px activity was observed by 42.5% in smokers and 58.5% in non-smokers. In an in vivo study, after 30 min, the activity level returned to 90-100% of the pre-smoking level due to the production of new saliva. The decreased Px activity was accompanied by increased carbonylation of salivary proteins. | Reznick et al., 2003) |
| Evaluating the influence of smoking on **Px** in human saliva. | Nonsmokers (n – no data) | **Unstimulated saliva** | A three-fold puff of CS caused a statistically significant decrease in Px activity by 76%. | Klein et al., 2003) |
| Investigating the effect of cigarette smoking on the parameters of salivary oxidative stress (reflected by **SOD** activity and **TAC** value). | The patients (n=50) were divided into two groups:  1. heavy smokers (n=25).  2. nonsmokers (n=25). | **Unstimulated saliva** | Smokers were characterized by a significantly higher value of the median SOD activity and TAC compared to the nonsmoking group. Moreover, smokers experienced a significant increase in the concentration of carbonyl in saliva and a significant decrease in the concentration of lactate dehydrogenase and metalloproteinases. | (Nagler, 2007) |
| Comparison of salivary **TAC** and **Vitamin C** in smokers and non-smokers | The patients (n=100) were divided into two groups:  1. active smokers (n=50).  2. nonsmokers (n=50). | **Unstimulated saliva** | TAC and vitamin C content in smokers saliva were significantly lower than in non-smokers saliva. | (Falsafi et al., 2016) |
| Investigating the effect of intense physical exercise on the activity of salivary antioxidant enzymes in smoker and nonsmoker  reflected by activity of **Px**, **CAT**, **SOD** measured at 3 different time points: pre-test (TP1), post-test (TP2), and one hour post-test end (TP3). | The patients (n=40) were divided into two groups:  1. Smoker exercising/SE (n=10).  2. Smokers not exercising/SnE (n=10).  3. Non-smoker exercising/NSE (n=10).  4. Non-smoker non-exercisers/NSnE (n=10). | **Unstimulated saliva** | Significant interactions between the group x time relationship for the three tested enzymes were found. The results of the study indicate that the activity of antioxidant enzymes at rest and during recovery from acute, strenuous exercise was lower in SE and SnE compared to NSE and NSnE, proving that the addiction to smoking may reduce the mitigating effect of regular physical activity on oxidation induced by strenuous exercise. | (Nobari et al., 2021 |
| Determining the effect of passive smoking on salivary **TAC** and **lipid peroxidation** levels of adolescents aged 12-15 years. | The patients (n=80) were divided into two groups:  1. passive smokers (n=40).  2. nonsmokers (n=40). | **Unstimulated saliva** | Passive smoking may reduce the salivary TAC of adolescents, there was a significant difference in TAC between the test group and the control group. There was no significant difference between the passive smoker group and the control group in the level of lipid peroxidation. | (Neshat et al., 2020) |
| Determining the influence of smoking on salivary **TAC** levels of young people. | The patients (n=40) were divided into two groups:  1. smokers (n=20).  2. nonsmokers (n=20). | **Unstimulated saliva** | There was no significant difference in the salivary TAC levels between the two groups. | (Charalabopoulos et al., 2004) |
| Evaluating the effect of smoking on salivary **TAC** levels. | The patients (n=60) were divided into two groups:  1. smokers (n=30).  2. nonsmokers (n=30). | **Unstimulated saliva** | Smoking changes the antioxidant capacity of saliva, leading to a significant decrease in TAC values in smokers compared to the control group. | (Bakhtiari et al., 2015) |
| Evaluating the influence of passive smoking on salivary **TAC** and **UA.** | The patients (n=90) were divided into two groups:  1. passive smokers (n=45).  2. nonsmokers (n=45). | **Unstimulated saliva** | There was no statistically significant difference in the antioxidant capacity between the studied groups. Uric acid concentration was significantly lower in the group of passive smokers. | (Azadbakht M et al., 2016) |
| Determining the effect of passive smoking on **TAC** and **lipid peroxidation products** in saliva. | The patients (n=60) were adolescents aged 12-15 years divided into two groups:  1. passive smokers (n=30).  2. nonsmokers (n=30). | **Unstimulated saliva** | Salivary TAC was significantly lower in the group of passively smoking adolescents compared to the control group. The level of salivary lipid peroxidation products was higher in passive smokers than in the group of non-smoking adolescents, however, the difference was not statistically significant. | (Mottalebnejad M et al., 2014) |
| Investigating the influence of vitamin C on salivary **TAC** level of smoking individuals. | Smokers (n=30) | **Unstimulated saliva** collected before vitamin C supplementation and after 3 weeks of supplementation. | The oxidative stress expressed by the level of TAC induced by cigarette smoke did not significantly decrease after 3 weeks of vitamin C supplementation. | (Bakhtiari et al., 2012) |
| Evaluating the influence of green tea drinking on changes in salivary **TAC** level of smokers and nosmokers. | The patients (n=40) were divided into two groups:  1. smokers (n=20).  2. nonsmokers (n=20). | **Unstimulated saliva** collected after 7 days and 3 weeks of green tea drinking. | On day zero, the controls had higher TAC levels than the smokers. There was an upward trend in both smokers and non-smokers during the study period (after tea drinking). There was a significant difference in TAC in smokers versus nonsmokers from baseline to day 21. | (Azimi et al., 2017) |
| Evaluating the effect of smoking and tea drinking habits on expression of ROS-related **NF-κB proteins** and **p53,MLH1 proteins** associated with DNA repair. | Exfoliated cheek cells were collected from 308 healthy subjects classified by age, smoking and tea drinking habits | Exfoliated **cheek cells** | Smokers, regardless of their age, were characterized by significantly higher ROS concentration and frequency of DNA damage than non-addicted people. Tea drinking was effective in reducing ROS levels, DNA damage, and IκB, NF-κB, p53 and MLH1 expression in tobacco users regardless of age. | (Pal et al., 2012) |
| Determining the differences in salivary **MDA** concentration between non/passive/active smokers. | The patients (n=60) were divided into two groups:  1. Non-smoker (n = 20)  2. Passive smokers (n = 20)  3. Active smokers (n = 20) | **Unstimulated saliva** | The concentration of MDA was significantly higher in the group of active smokers compared to the other two groups. In turn, passive smoking leads to a significantly higher lipid peroxidation expressed in MDA concentration compared to the control group. | (Demirtaş M et al., 2014) |
| Investigating the influence of smoking and periodontitis on **lipid peroxidation** and salivary oxidative stress parameters (reflected by **MDA**, **GPx** and **TAC** levels) | The patients (n=60) were divided into 4 groups:  1. healthy nonsmokers (n=15)  2. nonsmokers with periodontitis (n=15)  3. healthy smokers (n=15)  4. smokers with periodontitis (n=15) | **Unstimulated saliva** | Patients with periodontitis show higher lipid peroxidation, decreased GPx activity and TAC levels than healthy subjects. This effect is exacerbated by smoking. Non-surgical periodontal treatment reduces MDA and GPx to levels comparable to healthy controls. | (Guentsch et al., 2008a) |
| Evaluating the effect of smoking on levels of salivary **TAC**, **TOS**, **MDA**, **NO** and **GPx** activity. | The patients (n=54) were divided into 2 groups:  1. nonsmokers (n=27).  2. smokers (n=27). | **Unstimulated saliva** | Smokers have significantly higher levels of MDA before and after smoking. The activity of smokers' salivary GPx was significantly lower than in the control group, and the nitric oxide level in saliva after smoking was higher than in the control group, both before and after smoking a cigarette. | (Kurku H et al., 2015) |
| Determining the influence of smoking on levels of salivary **TAC**, **UA**, amylase and **LDH** as well as the effect of vitamin C on the above-mentioned parameters | Nonsmokers (n=6) | **Unstimulated saliva** | Oral exposure to CS leads to a statistically significant decrease in the activity of UA, LDH and salivary amylase. Addition of vitamin C had a significant protective effect on UA acid level. | (Greabu et al., 2007) |
| Determining and comparing the salivary **NO** levels in healthy nonsmoking patients with smokers, tobacco chewers and patients with oral lichenoid reactions. | The patients (n=120) were divided into 4 groups:  1. Healthy patients without chronic inflammation in the mouth and smoking addiction (n = 30).  2. Smokers without the habit of chewing tobacco and chronic inflammation in the mouth (n = 30).  3. People chewing tobacco, no smoking addiction and no change in the mouth (n = 30).  4. People with oral lichenoid reactions to smoking addiction (n=30). | **Unstimulated saliva** | Significant increases in NO levels have been demonstrated in smokers, people chewing tobacco, and smoking patients with oral lichenoid reactions compared to the control group. Significant increases in nitric oxide levels were also observed in patients with smoking-related lichenoid reactions compared to smokers, and in patients with lichenoid reactions related to tobacco-chewing compared to tobacco-chewing individuals. | (Preethi, 2016) |
| Investigating the effect of smoking and periodontitis on salivary lipid peroxidation and parameters of oxidative stress reflected by **MDA**, **GPx** and **TAC** levels. | The patients (n=60) were divided into four groups:  1. Healthy nonsmokers (n=15).  2. Nonsmokers with periodontitis (n=15).  3. Healthy smokers (n=15).  4. Smokers with periodontitis (n=15). | **Unstimulated saliva** | Patients with periodontitis show higher lipid peroxidation, decreased GPx activity and TAC levels than healthy subjects. This effect is exacerbated by smoking. Non-surgical periodontal treatment reduces MDA and GPx to levels comparable to healthy controls. | (Guentsch et al., 2008a) |
| Comparing the activity of **Cu/Zn SOD**, **P**x and **GPx** in the saliva of smokers and nonsmokers. | The patients (n=88) were elderly men divided into two groups:  1. nonsmokers (n=44).  2. smokers (n=44). | **Unstimulated saliva** | SOD activity was significantly higher in the smoking group than in the non-smoking group, while the levels of Px and GPx activity were significantly higher in the non-smoking group than in the smoking group. | (Kanehira et al., 2006) |
| Evaluating the effect of  expose to cigarette smoke to affinity the 18kDA translocator protein **(TSPO)** for its specific ligand as well as assessing **carbonyls and lipid peroxides.** | Healthy non-smoking ( n – no data) | **Unstimulated saliva** | After exposure of saliva to cigarette smoke, the affinity of TSPO in saliva for its specific ligands decreased threefold compared to the sham-treated control. The changes in the affinity of saliva TSPO were accompanied by an increase in the concentration of carbonyl and lipid peroxides. | (Nagler et al., 2010) |
| Assessing the salivary activities of **SOD, GPx** and **CAT** and **MDA** levels of smokers with periodotitis. | The patients (n=65) were divided into four groups:  1. chronic periodontal smokers [CP-S] (n=23).  2. chronic periodontal ex-smokers [CP-FS] (n=23).  3. chronic periodontal non-smokers [CP-NS] (n=19).  4. healthy controls (PH-NS) (n=20). | **Gingival tissue** samples | The decreased local SOD, GPx and CAT activity observed in patients with periodontitis may worsen with smoking. The levels of MDA in the gingival tissues in the periodontitis groups were significantly higher than in the control group. The control group had the highest SOD, GPx and CAT activity compared to all the periodontitis groups. The CP-S group had the highest levels of MDA in the gingiva and the activity of SOD, GPx and CAT among the periodontitis groups, while the lowest values were observed in the CP-NS group. MDA levels in the CP-FS group were similar in the CP-NS group, but lower than in the CP-S group. | (Tonguç et al., 2011) |
| Evaluating the effects of cigarette smoking and gingivitis on salivary **GSH**, **AC**, **TAC**  and whole mouth clinical periodontal parameters as well as comparing the results at baseline and one month after the initial treatment phase in patients with gingivitis. | The patients (n=40) were divided into four groups:  Group 1: 10 healthy non-smoker subjects (n=8).  Group 2: 10 healthy smoker subjects (n=12).  Group 3: 10 non-smoker gingivitis subjects (n=11).  Group 4: 10 smoker gingivitis subjects (n=9). | **Unstimulated saliva** | Smoking cigarettes have no effect on the antioxidant capacity of saliva in patients with gingivitis. The concentration of GSH in saliva was significantly lower after the treatment in patients with gingivitis who smoked cigarettes. There were no statistically significant differences between the groups in the biochemical parameters at the beginning / after the periodontal treatment. Both smokers and non-smokers improved the periodontal clinical parameters after periodontal treatment. | (Buduneli et al., 2006) |
| Assessing the **SOD** activity of smoking individuals. | The patients (n=70) were divided into two groups:  1. smokers (n=60).  2. nonsmokers (n=10). | **GCF** | There was a gradual decline in SOD levels from healthy nonsmokers to light smokers to heavy smokers. | (Agnihotri et al., 2009) |
| Evaluating the influence of smoking and periodontitis on salivary concentration of **MT**. | The patients (n=85) were divided into four groups:  1. chronic periodontitis smoker [CP + S] (n=23).  2. chronic periodontitis nonsmoker [CP + NS] (n=22).  3. healthy periodontitis smoker [PH + S] (n=20)..  4. healthy periodontitis nonmoker [PH + S] (n=20). | **Unstimulated saliva** | Smoking and chronic periodontitis, by increasing oxidative stress and increased intoxication with heavy metals, may indicate MT synthesis. MT levels in the CP + S group were significantly higher compared to the other three groups. There was no statistically significant difference in MT levels between the CP + NS and PH + S groups. | (Yadav et al., 2020) |
| Determining the influence of smoking on the development of periodontal disease by examining tissues for **MT** level. | The patients (n=33) with periodontitis were divided into four groups:  1. smokers (n=22).  2. nonsmokers (n=11). | **Tissues from gingival** biopsy | MT was detected in both smokers and non-smokers, however, in the group of smokers, the thoracic and basal epithelial layers had a significantly higher ratio of metallothionein-positive cells compared to nonsmokers. | (Katsuragi et al., 1997) |
| Evaluating the influence of smoking on salivary concentration of **MT**. | The patients (n=217) were divided into two groups:  1. smokers (n=113).  2. nonsmokers (n=113). | **Unstimulated saliva** | Smokers had higher levels of MDA and fewer epithelial cells in unstimulated saliva compared to non-smokers. | (Celec et al., 2005) |
| Investigating the influence of periodontitis with additional smoking for influence on the level of prostaglandins in saliva **8-epi-PGF(2α)** | The patients (n=121) with periodontitis were divided into two groups:  1. smokers (n=90).  2. nonsmokers (n=31). | **Unstimulated saliva** | Oxidative stress reflected in elevated levels of 8-epi-PGF(2α) in saliva, is associated with the prevalence of periodontal disease and is greatly exacerbated by concomitant tobacco abuse. The level of 8-epi-PGF (2alpha) was significantly higher in smokers compared to non-smokers. | (Wolfram et al., 2006) |
| Evaluating the influence of periodontitis with additional smoking as well as nonsurgical periodontal treatment on the level of **8-OHdG** | The patients (n=217) were divided into two groups:  1. smokers (n=113).  2. nonsmokers (n=113). | **Unstimulated saliva** | The baseline 8-OHdG level in saliva was highest in the periodontitis smokers group compared to the other groups. After non-surgical periodontal treatment, 8-OHdG values of periodontitis smokers were still higher than those of non-smokers. | (Wolfram et al., 2006) |
| Determining the influence of periodontitis with additional smoking as well as nonsurgical periodontal treatment on the level of salivary **8-OHdG, HNE** and **GPx** activity. | The patients (n=93) were divided into four groups:  1. chronic periodontitis smoker (n=24).  2. chronic periodontitis nonsmoker (n=23).  1. chronic periodontitis smoker (n=23).  3. healthy periodontitis smoker (n=23)..  3. healthy periodontitis nonmoker (n=23) | **Unstimulated saliva, GCF** collected and analyzed at the beginning and on the 1st and 3rd month after periodontal treatment. | The concentration of 8-OHdD in GCF is significantly higher in both groups with periodontitis compared to both groups of healthy periodontitis. 8-OHdG and GPx in saliva in both periodontitis groups were significantly increased compared to the non-periodontitis nonsmoking group. In the group of smokers with periodontal disease, the concentration of 4-HNE in GCF is significantly higher than in healthy periodontium. Initial periodontal therapy may be helpful in reducing oxidative stress in periodontitis. After initial periodontal treatment, the levels of 8-OHdG in GCF and saliva were significantly reduced in both periodontitis groups. | (Hendek et al., 2015) |
| **Experimental Study** | | | | |
| Investigating the influence of increasing puffs of cigarette smoke and assessing changes in **protein carbonylation** and **GSH** level on fibroblast cells. |  | **HGF** | Exposure of HGFs to cigarette smoke has been shown to reduce cell protein thiols and deplete intracellular GSH, with slight increases in intracellular levels of glutathione disulfide, S-glutathionyl proteins and glutathione levels. The decrease in GSH concentration may be a mechanism of cigarette smoke-induced cytotoxicity and may be correlated with the decreased repair and regenerative activity of gingival and periodontal tissues. | (Colombo et al., 2012) |
| Evaluating the influence 14C-testosterone / 14C-4-androstenedione in the presence or absence of nicotine, glutathione (G1-5) and combinations thereof to test the oxidative effect of nicotine. |  | **HGF** and **HPF** | Nicotine downregulated the physiologically active metabolite 5α-dihydrotestosterone in HGF and HPF, overregulated to varying degrees by the antioxidant glutathione. | (Tinti and Soory, 2012) |
| Determinig the influence of various concentrations of nicotine and P. gingivalis lysate on oxidative stress (expressed by the concentration of **MDA** and **Ox-GS**) after 2 and 24 hours. |  | **PDLF** | Apart from the levels of MDA in the P. gingivalis lysate at 20 µg / ml, the levels of MDA under all other conditions tested were the same as in the positive control at 24 hours. The production of ROS and superoxides was increased upon stimulation with P.gingivalis and / or nicotine. Therefore, OS biomarkers were generated by PDLF after treatment with periodontal pathogens and nicotine, which may explain a potential local mechanism of periodontal disease etiology through superoxide mediation. | (Nguyen et al., 2019) |
| Evaluating the influence of nicotine on the expression of HO-1 protein. Antioxidants **CAT, SOD** and **NAC** were added to cells incubated with nicotine to see how they modulate the effect on nicotine-induced HO-1 expression. |  | human **fibroblastcultures** and **gingival tissues** obtained by biopsy | The results indicate that the expression of HO-1 is significantly increased in the gingival tissues of cigarette smokers. Addition of GSH precursor NAC inhibited nicotine-induced expression of HO-1 protein. SOD and catalase were able to reduce the nicotine-induced expression of HO-1 protein. | (Chang et al., 2005) |
| Evaluating the effect of nicotine on the expression of the **COX-2** mRNA gene and protein. |  | **HGF** | Nicotine leads to increased activation of COX-2 expression. These results may explain the potential role of smoking in the pathogenesis of periodontal disease. Moreover, nicotine-induced cytotoxicity does not result directly from the induction of COX-2 expression. | (Chang et al., 2003c) |
| Determining the effect of systemic resveratrol administration (RESV) on oxidative stress during experimental periodontitis. | Wistar rats (n=26) divided into groups:  1.cigarette smoke inhalation (SMK) + RESV (n=13).  2. cigarette smoke inhalation + placebo (PLAC) (n = 13). | **Bone of rats.**  and **gingival tissues** surrounding the mandibular first molars. | A significant reduction in bone loss was demonstrated in the SMK + RESV group compared to the SMK + PLAC group. Resveratrol caused significantly higher SOD activity and decreased levels of NADPH oxidase in tissues from animals in the SMK + RESV group compared to those in the SMK + PLAC group. | (Corrêa et al., 2019) |
| Determining the genotoxicity and mechanisms induced by EC aerosol extracts **(DNA damage, ROS concentration and TAC levels )** |  | human **oral epithelial cells** | Exposure to EC aerosol extracts suppressed cellular antioxidant defense mechanisms (increased ROS, decreased TAC, and 8-oxoguanine DNA glycosylase expression) and led to significant DNA damage. | (Ganapathy et al., 2017) |
